# Plant Growth-Promoting Effect and Complete Genomic Sequence Analysis of the Beneficial Rhizosphere *Streptomyces* sp. GD-4 Isolated from *Leymus secalinus*

**DOI:** 10.3390/microorganisms13020286

**Published:** 2025-01-27

**Authors:** Wanru Xu, Yimeng Liu, Yiping Cheng, Jie Zhang

**Affiliations:** Key Laboratory of Biological Resources and Ecological Environment of Ministry of Education, College of Life Sciences, Sichuan University, Chengdu 610064, China; xuwanru@stu.scu.edu.cn (W.X.); liuyimeng@stu.scu.edu.cn (Y.L.); chenyiping@stu.scu.edu.cn (Y.C.)

**Keywords:** plant growth promotion, *Streptomyces*, genome analysis, dissimilatory nitrate reduction to ammonium, alpine grassland restoration

## Abstract

Plant growth-promoting rhizobacteria (PGPR) are beneficial bacteria residing in the rhizosphere and are capable of enhancing plant growth through various mechanisms. *Streptomyces* sp. GD-4 is a plant growth-promoting bacterium isolated from the rhizosphere soil of *Leymus secalinus*. To further elucidate the molecular mechanisms underlying the beneficial effects of the strain on plant growth, we evaluated the growth-promoting effects of *Streptomyces* sp. GD-4 on forage grasses and conducted comprehensive genome mining and comparative genomic analysis of the strain. Strain GD-4 effectively colonized the rhizosphere of three forages and significantly promoted the growth of both plant roots and leaves. Genome sequence functional annotation of GD-4 revealed lots of genes associated with nitrogen, phosphorus, and sulfur metabolism. Additionally, genes potentially involved in plant growth promotion such as indole-3-acetic acid (IAA) biosynthesis, trehalose production, siderophore production, and phosphate solubilization were annotated. Whole-genome analysis revealed that GD-4 may possess molecular mechanisms involved in soil nutrient cycling in rhizosphere soil and plant growth promotion. The bacteria also possess genes associated with adaptability to abiotic stress conditions, further supporting the ability of *Streptomyces* sp. GD-4 to colonize nutrient-poor soils. These findings provide a foundation for further research into soil remediation technologies in plateau regions.

## 1. Introduction

The ecological quality of the ecological environment in the Qinghai–Tibet Plateau region has an indicative effect on the global climate. Zoige is located in the hinterland of the Qinghai–Tibet Plateau and is an important water conservation area for the Yellow River and Yangtze River [1]. Due to nearly 40 years of overgrazing, some grasslands in the Zoige area have become desertified and soil fertility has been severely depleted [2]. Numerous studies have shown that the soil fertility of Zoige degraded sandy land is far lower than that of normal grassland; in particular, the nitrogen content is dozens of times different than that of normal grassland soil [3]. It is difficult for most replanted grasses to colonize and grow in the sandy soil. The pioneer plant *Leymus secalinus* is a native perennial herbaceous plant in alpine grasslands [4]. As an important plant to resist desertification, it can grow in severely desertified soil, thanks to its rapid growth and well-developed root system. Through previous rhizosphere metagenomic studies [4,5], we speculated that in hotspots of microbial activity, rhizosphere microorganisms play an indispensable role in the colonization process of pioneer plants. Plant growth-promoting rhizobacteria (PGPR) can promote plant growth through a variety of mechanisms, including increasing the absorption of nutrient uptake [5], the production of plant hormones [6], resistance to biotic and abiotic stress, and the promotion of improvement of the rhizosphere environment [7]. However, for plants in plateau and poor-soil areas, limited nutrients, extreme climate, and high altitude will limit the effects of these mechanisms. Therefore, isolating plant growth-promoting rhizobacteria from native plants can effectively harness adaptation to harsh soil conditions.

In areas experiencing grassland desertification, nitrogen typically originates from atmospheric deposition [8], animal urine [6], and the microbial nitrogen cycle [9]. The nitrogen required for restoring degraded grasslands typically takes a prolonged accumulation period to support sufficient plant growth. To enhance plant growth and colonization efficiency, improve soil fertility, and leverage the growth of pioneer plants to combat desertification, it is essential to explore strategies for increasing nitrogen content in the soil [10]. From the perspective of plant growth, the absorption and conversion of existing inorganic nitrogen sources are critical. Therefore, utilizing the interactions between plants and rhizosphere microorganisms is key to improving nitrogen use efficiency [11,12,13].

Grassland degradation is often accompanied by nitrogen loss, which may become an important condition limiting the growth of pioneer plants [14]. For instance, nitrogen sources available to plants include both direct and indirect forms, such as nitrate and ammonium. Ammonium can be directly absorbed and assimilated by plants, while nitrate typically enters the plant and is subsequently converted into ammonium [15]. However, studies have shown that nitrate in sandy soils is prone to loss through leaching or denitrification, which converts it into gaseous nitrogen, whereas ammonium nitrogen tends to be more readily retained in the soil [16]. As a result, the challenge in Zoige’s restored sandy lands, which are deficient in nitrogen sources, is to find effective strategies for nitrogen retention that can promote plant growth in these nitrogen-limited environments.

The nitrogen cycle of soil microorganisms typically encompasses six distinct nitrogen transformation processes [17]: nitrogen fixation, assimilation, ammonification, nitrification, denitrification, and anaerobic ammonium oxidation. Dissimilatory nitrate reduction to ammonium (DNRA) refers to the ability of certain microorganisms to utilize electron donors in the process of reducing nitrate to ammonium [18]. Numerous potentials remain to be explored in the current research on DNRA bacteria. Previous studies highlighted that the pathway of the dissimilatory reduction of nitrate to ammonium can preserve bioavailable nitrogen in soils deficient in organic matter [13,19]. Related reports from metagenomic research indicate that *Miscanthus condensatus*, a perennial grass species, serves as the primary pioneer plant in the acidic volcanic sediments of Miyake Island, Japan. In conditions characterized by soil nitrogen deficiency, a significant number of nitrogen cycle-related genes associated with DNRA (nirB, nirD) are present in its rhizosphere [12]. To restore the nitrogen balance affected by production imbalances, utilizing microorganisms for nitrogen conversion presents a sustainable solution [20]. Many articles currently emphasize the abundance of nitrogen cycle-related genes from microbial communities. For instance, there are reports utilizing metagenomic sequencing technology to investigate how the rhizosphere of alpine coniferous forests enhances plants’ acquisition of ammonium ions through the activities of rhizosphere microorganisms [11]. However, there are few relevant literature reports on the DNRA bacteria that promote growth from the rhizosphere of pioneer plants. To improve the practical application of DNRA bacteria in production, it is essential to identify and utilize superior bacterial strain resources.

In this study, we isolated growth-promoting bacteria from the roots of *Lycium chinensis* and obtained an *Actinomycete* strain. Following screening, culture, and whole-genome sequencing, we discovered that this strain has functional genes related to plant growth promotion. The annotation results of functional genes related to the nitrogen cycle indicated that the strain had the potential to dissimilate nitric acid to ammonium. Through pot experiments, it was observed that the subject exhibits a growth-promoting effect on various dominant grassland plants, including *Lolium perenne* L., *Elymus dahuricus* Turcz., and *Elymus sibiricus* L.

## 2. Materials and Methods

### 2.1. Sample Collection

Samples were collected from Axi Township (33°41′0″ N, 102°56′7″ E), Zoige County, Sichuan Province, where the average annual temperature ranges from 0.6 °C to 1.2 °C, and the average annual precipitation is between 600 mm and 800 mm [21]. For each 5 m × 5 m plot, 10 plants from similar growth patterns were randomly selected. The top 10 cm of soil was removed with sterilized shovels, and plants were carefully dug out. The roots were shaken to remove loosely attached soil, and the firmly adhered soil was collected as rhizosphere soil using a sterile brush. After cleaning and collecting the sandy soil attached to the plant root system, the soil was transplanted into a pot for storage. 

### 2.2. Isolation and Subculture of Bacteria

We weighed 10 g of *Leymus secalinus* root soil into a 250 mL Erlenmeyer flask and added 90 mL of sterile water. We then placed the flask in a constant-temperature shaking incubator set to 28 °C and to shake it at 120 rpm for 20 min. We allowed the mixture to settle and carefully collected the supernatant, representing the 10^−1^ dilution. Serial dilutions were then performed to obtain 10^−2^, 10^−3^, and 10^−4^ dilutions. From each dilution, 50 μL was spread onto nitrogen-free solid medium (Ashby) plates. Each concentration gradient was plated in triplicate. The plates were incubated at 28 °C for 3 to 5 days, during which strains exhibiting rapid growth were selected and subjected to streak purification. The purification process was repeated 2 to 3 times to isolate pure single colonies.

### 2.3. Plant Growth-Promoting Assay of Streptomyces sp. GD-4

We selected three grass species native to the local grassland (*Lolium perenne* L., *Elymus dahuricus* Turcz., and *Elymus sibiricus* L.) for a growth promotion experiment using potted plants. Before plant inoculation, the collected repaired grassland sandy soil was sieved and sterilized by autoclaving at 121 °C for 30 min. The sterilized plant seeds were germinated in the dark and subsequently transplanted into pots in a greenhouse for 2 days after one week. A bacterial suspension of *Streptomyces* sp. GD-4 was prepared. Once the plants had established stable growth in the pots, 5 mL of the actinomycete bacterial suspension (OD = 0.1) was injected via root irrigation, followed by appropriate watering for continued cultivation. After 30 days, measurements were taken for root length, leaf length, above-ground and under-ground biomass, root absorption area, and plant chlorophyll content.

### 2.4. Root Colonization of Streptomyces sp. GD-4

Strain GD-4 was cultured for 14 days at 28 °C using the slide culture method. The coverslips with attached hyphae were fixed in 5% glutaraldehyde, followed by dehydration through a graded series of ethanol solutions (30%, 50%, 70%, 90%, 95%, and absolute ethanol) [21]. After the initial sample preparation, the specimens were sputter-coated with a thin layer of gold–palladium, and their morphological characteristics were then observed using a scanning electron microscope (SEM). The observation method for the experimental group followed the procedure described above. Plant roots inoculated with bacteria were carefully excised from the pots and thoroughly rinsed with sterile water until no soil remained on the root surfaces. The roots were then fixed in 5% glutaraldehyde for 8 h, after which 1–2 cm segments of the main root were cut. After dehydration and drying in a graded ethanol series, the samples were examined using a scanning electron microscope (SEM) to assess microbial colonization on the root surfaces. The control group of plants was processed and imaged using the same procedure.

### 2.5. Ammonia Production

Ammonia production of the test strains was tested in peptone water (g/L: peptone 10.0; sodium chloride 5.0). Fresh culture (48 h age) was inoculated into 50 mL of peptone water and cultivated at 28 °C, 150 rpm, for 7 days. Nessler’s reagent (0.5 mL) (Macklin, Shanghai, China) was added to each bacterial suspension. Development of golden yellow color was noted as a positive result for ammonia production.

### 2.6. Comparative Genome Analysis

The average nucleotide identity (ANI) values among 11 genome sequences, including *Streptomyces* sp. GD-4 and other 10 *Streptomyces* strains, were calculated using the Majorbio online service [22]. ANI results were used for hierarchical cluster analysis using MUMmer v3.23 software.

### 2.7. Library Construction and Genome Sequencing

Genomic DNA was sequenced using a combination of PacBio Sequel IIe and Illumina sequencing platforms. For Illumina sequencing, genomic DNA was used for each strain in sequencing library construction. DNA samples were sheared into 400–500 bp fragments using a Covaris M220 Focused Acoustic Shearer following the manufacturer’s protocol. Illumina sequencing libraries were prepared from the sheared fragments using the NEXTFLEX Rapid DNA-Seq Kit (Illumina, San Diego, CA, USA). Briefly, 5′ prime ends were first end-repaired and phosphorylated. Next, the 3′ ends were A-tailed and ligated to sequencing adapters. The third step was to enrich the adapters-ligated products using PCR. The prepared libraries were then used for paired-end Illumina sequencing (2 × 150 bp) on an Illumina Novaseq 6000 (Illumina Inc., San Diego, CA, USA).

For PacBio sequencing, genomic DNA was fragmented at ~10 kb. The fragmented DNA was then purified, end-repaired, and ligated with SMRT bell sequencing adapters following the manufacturer’s recommendations (Pacific Biosciences, Menlo Park, CA, USA). Next, the PacBio library was prepared and sequenced on one SMRT cell using standard methods.

### 2.8. Genome Assembly and Annotation

The data generated from the PacBio Sequel IIe and Illumina platforms were used for bioinformatics analysis. The detailed procedures are as follows.

The raw Illumina sequencing reads generated from the paired-end library were subjected to quality filtering using fastp v0.23.0. HiFi reads were generated from the PacBio platform for analysis. Then, the clean short reads and HiFi reads were assembled to construct complete genomes using Unicycle v0.4.8 [23] and Pilon v1.22 to polish the assembly using short-read alignments, reducing the rate of small errors. The final assembled genome was submitted to the NCBI database (accession number: PRJNA1200240). The coding sequences (CDs) of chromosomes and plasmids were predicted using Glimmer or Prodigal v2.6.3 and GeneMarkS [24], respectively. tRNA-scan-SE (v2.0) [25] was used for tRNA prediction, and Barrnap v0.9 (https://github.com/tseemann/barrnap (accessed on 1 December 2024)) was used for rRNA prediction. The predicted CDs were annotated from NR, Swiss-Prot, Pfam, GO, COG, KEGG, and CAZY databases using sequence alignment tools such as BLAST, Diamond, and HMMER. Briefly, each set of query proteins was aligned with the databases, and annotations of best-matched subjects (e-value < 10^−5^) were obtained for gene annotation. Biosynthetic gene clusters (BGCs) of secondary metabolites were identified by antiSMASH v5.1.2 software.

## 3. Results

### 3.1. SEM Observation of the Morphology and Colonization of Strain GD-4

**Figure 1:**
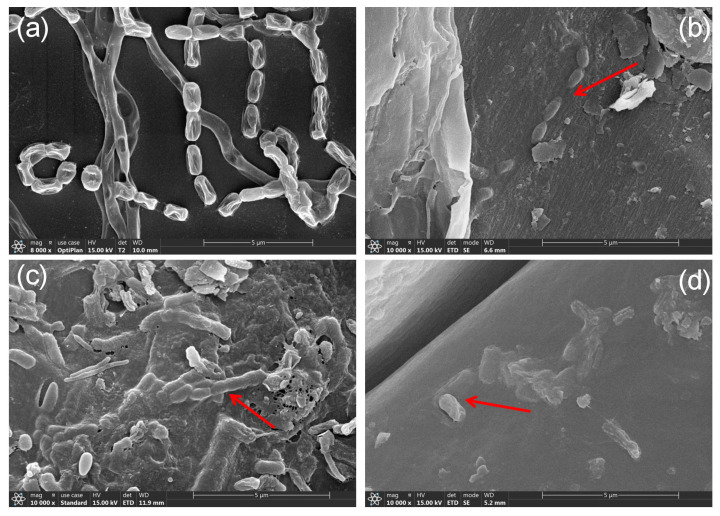
The observation of bacterial morphology and colonization by scanning electron microscopy (SEM), The red arrow indicates *Streptomyces* sp. GD-4. (**a**) Scanning electron micrograph of *Streptomyces* sp. GD-4 cells. (**b**) The root colonization of *Elymus dahuricus* Turcz. after inoculation of GD-4 by SEM. (**c**) The root colonization of *Lolium perenne* L. after inoculation of GD-4 by SEM. (**d**) The root colonization of *Elymus sibiricus* L. after inoculation of GD-4 by SEM.

*Streptomyces* sp. GD-4 was isolated from the rhizosphere of the pioneer plant *Leymus secalinus* and has demonstrated the ability to grow in nitrogen-deficient media (Ashby). Scanning electron microscopy (Figure 1) revealed that strain GD-4 exhibits the typical morphological characteristics of *Streptomyces*. SEM images taken after culturing on medium for 14 days show that GD-4 has highly branched hyphae. The hyphae in the culture medium formed curved spiral spore chains, and the free spores were smooth and rod-shaped (Figure 1a). The shrinkage of the bacterial cells observed in the electron microscope may be related to the dehydration treatment used in the experiment. Scanning electron microscopy observations of *Streptomyces* colonization in the roots of three grass species revealed clear colonization traces of *Streptomyces* sp. GD-4 in the rhizosphere of *Elymus dahuricus* Turcz. (Figure 1b), *Lolium perenne* L. (Figure 1c), and *Elymus sibiricus* L. (Figure 1d). Colonization was primarily concentrated in the taproot region. SEM observations revealed that *Streptomyces* sp. GD-4 formed a dense colonization structure on the main root of grass plants, closely interacting with the root surface through hyphae and extracellular secretions.

### 3.2. Effect of Strain GD-4 Inoculation on the Physiological Index of Plants

**Figure 2:**
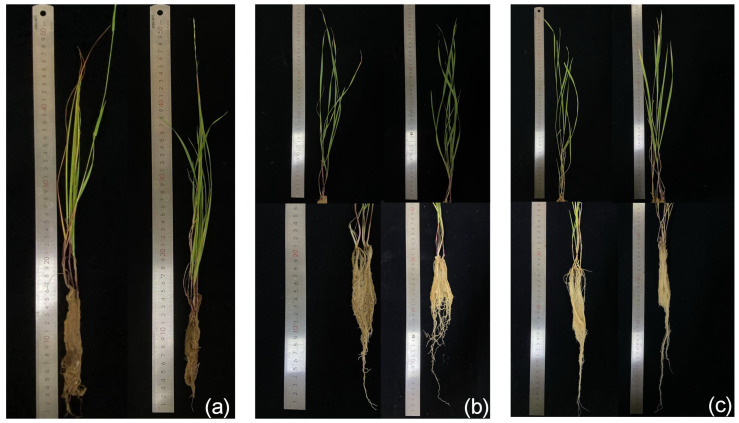
Plant growth promotion assay of *Streptomyces* sp. GD-4 on pasture after 30 days of inoculation. (**a**) From left to right: the *Lolium perenne* L. control group and the GD-4 inoculation group. (**b**) From left to right: the *Elymus sibiricus* L. control group and the GD-4 inoculation group. (**c**) From left to right: the *Elymus dahuricus* Turcz control group and the GD-4 inoculation group.

Greenhouse pot experiments were conducted using three grass species in degraded sandy soil. The results indicated that *Streptomyces* sp. GD-4 had varying degrees of growth-promoting effects on the development of gramineous plants under oligotrophic conditions (Figure 2).

**Figure 3:**
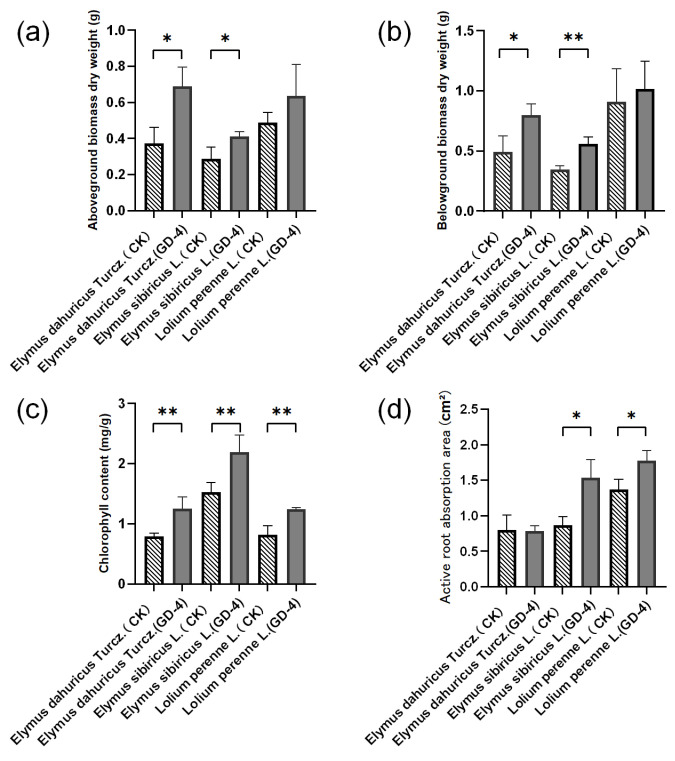
Effects of *Streptomyces* sp. GD-4 strain on growth parameters of different pasture species cultured in sandy soil for 30 Days: (**a**) leaf dry weight; (**b**) root dry weight; (**c**) chlorophyll content index. (**d**) Active root absorption area. The values represent the means of replicates (*n* = 3) ± standard deviations. Asterisks in superscript indicate a significant difference from the control at 95% between treatments. Each data point is the average of three replicates, and error bars represent ±SD. * Significance at *p* < 0.05; ** significance *p* < 0.01.

**Figure A1:**
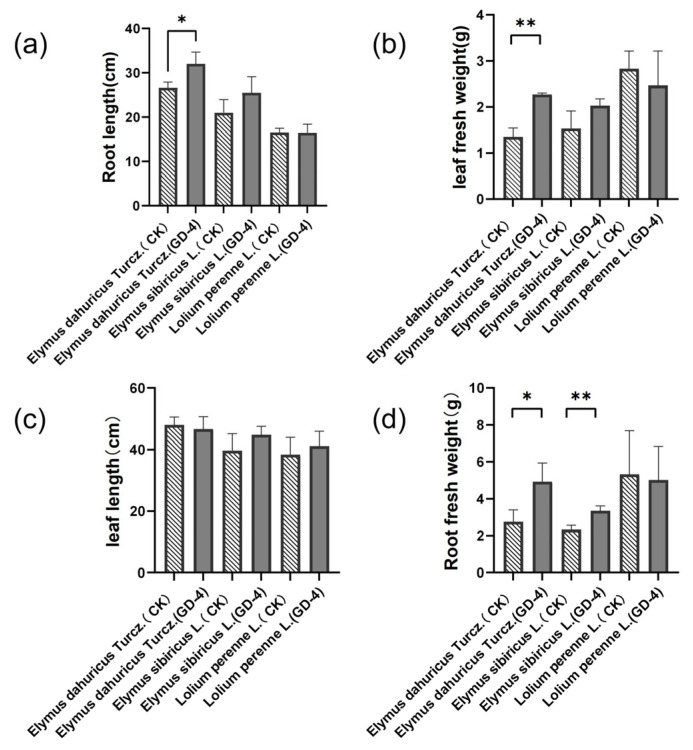
Effects of *Streptomyces* sp. GD-4 strain on growth parameters of different pasture species cultured in sandy soil for 30 days: (**a**) root length; (**b**) leaf fresh weight; (**c**) leaf length; (**d**) root fresh weight. The values represent the means of replicates (*n* = 3) ± standard deviations. Asterisks in superscript indicate a significant difference from the control at 95% between treatments. Each data point is the average of three replicates, and error bars represent ±SD. * Significance at *p* < 0.05; ** significance at *p* < 0.01.

Both the above-ground and below-ground biomass of the plants showed significant increases (Figure 3), particularly in the below-ground biomass of *Elymus sibiricus* L. and *Elymus dahuricus* Turcz (Figure 3b). Inoculation with strain GD-4 significantly increased the root length of *Elymus dahuricus* Turcz., and the root length and leaf length of *Elymus sibiricus* L. showed an increasing trend, but it was not statistically significant (Figure A1). In addition, inoculation with GD-4 significantly increased the chlorophyll content of the leaves of three plants (Figure 3c), and the active root absorption areas of two plants were increased (Figure 3d).

### 3.3. Comparative Genomics and Phylogenetic Analysis

**Figure 4:**
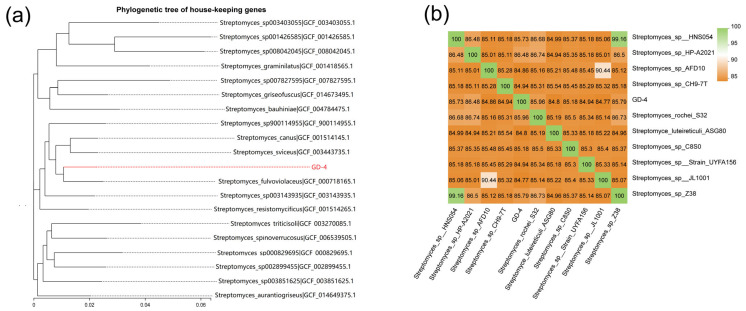
(**a**) Phylogenetic tree constructed based on 31 housekeeping genes using the Neighbor-Joining (NJ) method in MEGA 6.0 software. The red line represents *Streptomyces* sp. GD-4. (**b**) The heat maps of ANI (average nucleotide identity) between strain GD-4 and other 10 *Streptomyces* genus.

Phylogenetic analysis based on 31 housekeeping gene sequences (Figure 4) indicated that strain GD-4 is classified within the genus *Streptomyces*. It shares the highest sequence similarity with *Streptomyces canus* (95.9%) and exhibits the closest genetic relationship with *Streptomyces fulvoviolaceus* (96.2%). The strain forms a distinct clade with a bootstrap support value of 93.8%. However, since strain GD-4 could not be identified at the species level, it was designated as *Streptomyces* sp. GD-4. Although GD-4 shares many similarities with several *Streptomyces* species, there have been no reports on the growth-promoting mechanisms of this bacterium in the roots of pioneer plants in alpine sandy soils. The successfully assembled GD-4 genome was analyzed by average nucleotide identity (ANI) with 10 *Streptomyces* species (Table A1). The results showed that the ANI values for all 11 *Streptomyces* strains, including GD-4, were less than 95%; *Streptomyces* sp. HP-A2021 has the highest ANI value of 86.48%, followed by *Streptomyces rochei* S32 with 85.96%.

### 3.4. Genome Characteristics of Strain GD-4

**Figure A2:**
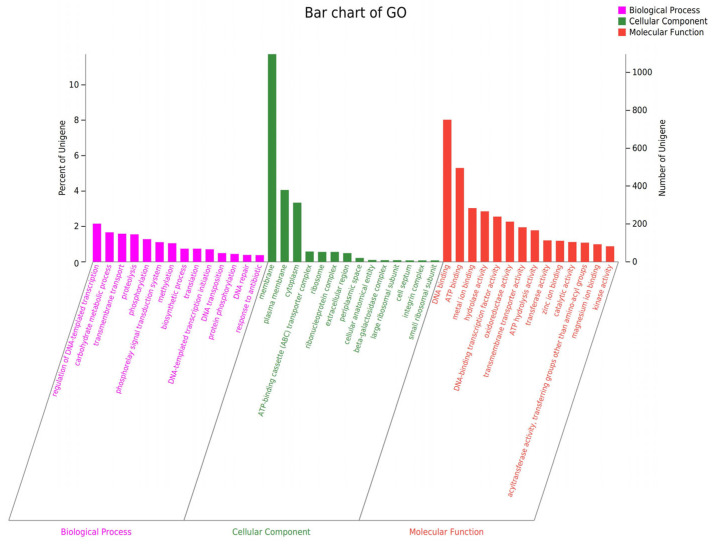
Classification of *Streptomyces* sp. GD-4 based on GO database annotation.

To further elucidate the mechanism by which bacterial GD-4 promotes the growth of pioneer plants under oligotrophic stress conditions, we analyzed its entire genome. We deposited the sequence information in NCBI GenBank. The analysis revealed that the chromosome length of strain GD-4 is 9,994,786 bp, with an average G + C content of 70.46% (Table 1). The complete GD-4 genome is estimated to contain 9352 coding sequences (CDSs), 18 rRNA genes, 71 tRNA genes, 62 sRNA genes (Table 1), a phage region, and a plasmid. A total of 9352 coding sequences (CDSs) were predicted. The Gene Ontology (GO), Clusters of Orthologous Groups (COG), and Kyoto Encyclopedia of Genes and Genomes (KEGG) databases were used to predict and annotate the GD-4 genes, resulting in 5435 (63.66%), 7009 (74.9%), and 6107 (65.3%) annotations, respectively (Table 2). GO annotations (Figure A2) revealed involvement in Biological Processes (2727), Cellular Components (2187), and Molecular Functions (4879). The major categories include integral components of membranes (1534), DNA binding (769), and ATP binding (539).

**Figure 5:**
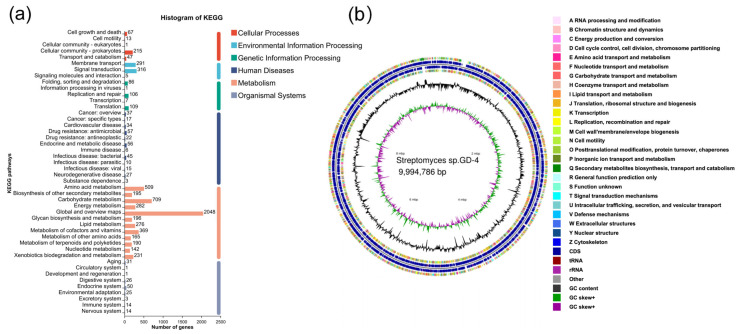
Classification of *Streptomyces* sp. GD-4 based on KEGG database annotation. (**a**) The ordinate indicates the level2 classification of the KEGG pathway, and the ordinate indicates the number of genes annotated under that classification. The column colors represent the level1 classification of the KEGG pathway. The right-most column shows the number of genes in different level1 categories. (**b**) Circular genome map of strain *Streptomyces* sp. GD-4. From the outer circle to the inner circle: The first and fourth rings represent the coding sequences (CDSs) on the forward and reverse strands. The second and third rings show the distribution of CDSs, tRNA, and rRNA on the positive and negative strands, respectively. The fifth ring depicts the GC content, while the sixth ring displays the GC-skew values.

**Figure A3:**
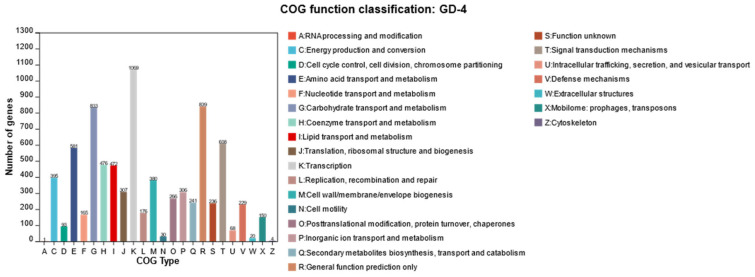
Classification of *Streptomyces* sp. GD-4 based on COG database annotation.

A total of 7009 COG-annotated genes were classified into 4 categories (Figure A3) and 24 distinct COG types. The most abundant functional clusters identified include Carbohydrate Transport and Metabolism (830 genes), Transcription (1071 genes), General Function Prediction Only (830 genes), Signal Transduction Mechanisms (610 genes), and Amino Acid Transport and Metabolism (581 genes). The KEGG database annotation identified six major pathway categories (Figure 5a): Cellular Processes (217 pathways), Metabolism (5495 pathways), Genetic Information Processing (275 pathways), Human Diseases (210 pathways), and Environmental Information Processing (412 pathways).

### 3.5. Genes Associated with Plant Growth Promotion in GD-4 Genome

Functional analysis of the GD-4 genome revealed the presence of multiple genes involved in various plant growth-promoting (PGP) functions, including nitrogen metabolism, sulfur metabolism, auxin and siderophore biosynthesis, phosphate solubilization, root colonization, and abiotic stress tolerance (Table 3 and Table 4).

#### 3.5.1. Nitrogen Metabolism

The ammonium production assay proved that GD-4 could produce ammonium nitrogen. In the prediction of nitrogen cycle genes in GD-4, genes related to the dissimilatory nitrate reduction to ammonium (DNRA) pathway were identified. Key annotations included genes encoding nitrate reductase (narH), nitrite reductase large subunit (nirB), nitrite reductase small subunit (nirD), and genes for related regulatory factors and molybdenum cofactors (mobA, moeB, and moaACDE) (Table 3). In addition, some genes related to nitrogen metabolism were identified. For instance, genes involved in the regulation of the nitrogen cycle include gltD (glutamate synthase small-subunit protein), gltB (glutamate synthase large-subunit protein), glnA (glutamine synthetase), and glnD (bifunctional nitrogen sensor protein) [26].

Among the genes related to nitrogen metabolism, we also identified genes involved in nitrogen transport, including nitrate/nitrite transporter (nark) and ammonium transporter (amt) [27]. Additionally, core genes involved in the regulation of nitrogen metabolism in prokaryotes were annotated, including those encoding nitrogen regulatory protein P-II 1 (glnB) and the related genes glnAED (Table A2). P-II proteins are multifunctional regulators in bacteria, archaea, and plastids, controlling nitrogen and carbon metabolism, transporters, and signaling molecules [28]. In addition, the gene NarL (nitrate/nitrite response regulator) is also present, typically involved in transcriptional activation as a nitrate response regulator in *E. coli* [29]. At the same time, genes related to nitric oxide reductase-activating proteins (NorD, NorQ) have not been identified [30].

Additionally, among the genes implicated in nitrogen metabolism in the GD-4 genome, the gene encoding the urea transport system permease protein (urtC) and its associated urtABDE gene cluster were identified (Table 3). These genes play a crucial role in the efficient transport of extracellular urea into the cell, thereby facilitating the acquisition of a stable nitrogen source from animal urine under nitrogen-limited conditions.

#### 3.5.2. Sulfur Metabolism

The GD-4 genome contains the gene clusters cysJHCDN and ssuABC, which are involved in sulfur metabolism and sulfate transport. These clusters encode key enzymes such as adenylylsulfate kinase (cysC), sulfite reductase (cysJ), and phosphoadenosine phosphosulfate reductase (cysH). Genes related to transport include those encoding 3-mercaptopyruvate sulfurtransferase (sseA). Through the action of the ssuABC gene cluster, GD-4 is capable of converting organic sulfides and sulfates into H_2_S. Studies have demonstrated that exogenous H_2_S, acting as a signaling molecule, responds to environmental stresses such as heavy metals, drought, and salinity, thereby regulating plant growth [31,32,33]. The tau family genes (tauA, tauB, tauC, tauD) are involved in the thiophenylalanine (taurine) metabolic pathway, assisting GD-4 in utilizing sulfur from taurine to meet its sulfur requirements. The genome annotation revealed various sulfur ABC transporters, membrane proteins, and the cysteine desulfurase subfamily, which includes sulfur carrier proteins (thiQ, thiP, thiB, thiE, and thiS). Additionally, cysteine desulfurase (sufS) and the Fe-S cluster assembly protein gene cluster (sufBCD) were also identified.

#### 3.5.3. Phosphate Solubilization

The GD-4 genome contains genes involved in phosphate transport and assimilation, including the phosphate transport system substrate-binding protein (pstS), ATP-binding protein (pstB), permease protein (pstAC), and alkaline phosphatase (phoAD). Additionally, it harbors genes encoding inorganic pyrophosphatase (PPA) and the phosphate metabolism regulatory gene exopolyphosphatase/guanosine-5′-triphosphate,3′-diphosphate pyrophosphatase (ppx-gppA). The GD-4 genome also includes the glucose 1-dehydrogenase (gdh) gene, which aids in the oxidation of glucose to synthesize GA and is involved in the regulation of phosphate (Table A3). However, the gene encoding the cofactor pyrroloquinoline quinone (PQQ) for GA production has not been annotated. Additionally, the genome contains genes for phosphonoacetate (phnA), phosphate transport, binding proteins, and sensing and signal transduction genes (phoUHP).

#### 3.5.4. Auxin Biosynthesis

**Figure 6:**
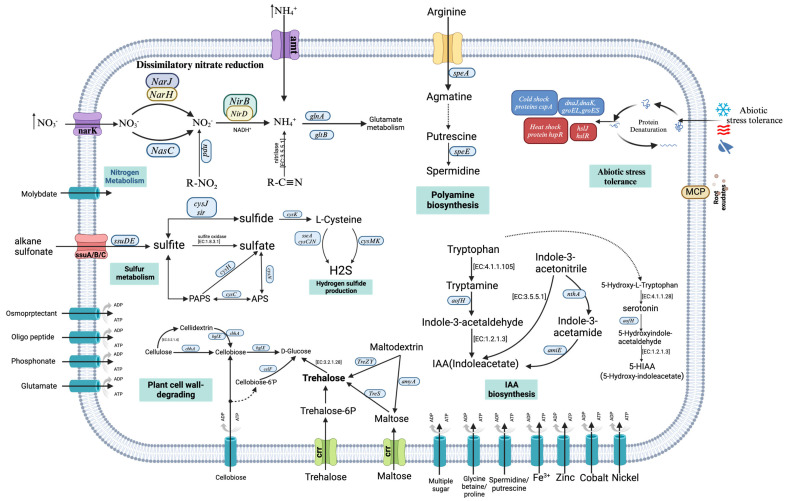
Schematic overview of metabolic pathways and transport systems in *Streptomyces* sp. GD-4. Individual pathways are denoted by single-headed arrows, while reversible pathways are denoted by double-headed arrows. Dashed arrows represent genes missing in genomes. The figure was created with BioRender.com.

The GD-4 genome has three annotated tryptophan-dependent synthesis pathway complete or partial genes, IAM, IAN, and TAM Pathway, in which indole-3-acetaldehyde serves as an intermediate from tryptophan through L-tryptophan decarboxylase [EC:4.1.1.105] and monoamine oxidase (aofH) and is catalyzed by aldehyde dehydrogenase [EC:1.2.1.3] to finally generate IAA (Figure 6). In the other two pathways, IAN is formed by indole-3-acetonitrile catalyzed by nitrilase [EC:3.5.5.1], and TAM is formed by indole-3-acetamide catalyzed by the enzyme encoded by gene amiE (Table A4). At the same time, indole-3-acetonitrile can also form indole-3-acetamide through the enzyme encoded by the gene nthAB (nitrile hydratases) (Table 4) and finally generate IAA [34]. Tryptophan synthesis genes are annotated in the GD-4 genome, and tryptophan (Trp) is a general precursor for the synthesis of IAA by most bacteria. Among them, the tryptophan operon (trpABCDE) generates L-tryptophan through the catalysis of a series of enzymes and enters the IAA synthesis pathway.

#### 3.5.5. Identification of Genes Responsible for Bacterial Biocontrol

*Streptomyces* serve as a rich source of natural antibiotics and related compounds, and they are extensively studied in both agricultural and medical fields [35]. The GD-4 genome contains genes involved in the synthesis of phenazine, enediyne antibiotics, ansamycins, vancomycin, and tetracycline (Table A5). For example, the phzEFS genes that are reported to regulate phenazine biosynthesis are annotated in GD-4 [36].

### 3.6. Abiotic Stress Tolerance

The ectB gene (betaine-aldehyde dehydrogenase) annotated in GD-4 plays a role in responding to environmental stress conditions, such as high salinity or drought, by promoting the accumulation of ectoine. Simultaneously, the betIBA operon encodes a group of enzymes regulated by the betI gene. This operon facilitates the conversion of choline into glycine betaine, enabling bacteria to adapt to osmotic stress [37]. The groEL and groES genes encode molecular chaperones that assist cells in managing heat stress. Additionally, the cspA gene, encoding a cold-shock protein (CspA), was identified in GD-4, while the hspR gene, a heat-shock protein transcriptional regulator, plays a role in modulating stress responses. The hslJ, hslR, dnaJ, and dnaK genes are involved in protein folding and repair processes [38].

Additionally, genes involved in oxidative stress response, including gamma-glutamyltranspeptidase (ggt), glutathione S-transferase (gst), and thioredoxin reductase (trxAB), were identified. Genes related to salt stress, such as proABPSVWX, which encodes the proline transport system substrate-binding protein, were also predicted. Furthermore, genes associated with trehalose synthesis, including isoamylase (TreZ), trehalose 6-phosphate synthase (OtsA), trehalose 6-phosphate phosphatase (OtsB), and maltose alpha-D-glucosyltransferase (TreS), were annotated.

### 3.7. Specific Gene Clusters in Streptomyces sp. GD-4

AntiSMASH analysis of the GD-4 genome identified 25 gene clusters associated with secondary metabolite biosynthesis on the chromosome and 2 additional clusters on the plasmid. These include four clusters encoding non-ribosomal peptide synthetases (NRPSs), three NRPS-like clusters, four siderophore clusters, six terpene clusters, two type I polyketide synthase (T1PKS) clusters, two melanin clusters, and seven clusters associated with other secondary metabolites (Table 5).

The secondary metabolites predicted from the chromosomal gene clusters are primarily antibiotics, siderophores, and terpenes. Salinomycin and chlorinated polyketides, encoded by plasmid gene clusters, along with istamycin, synthesized by chromosomal gene clusters, are all associated with antibacterial activity [39]. Naphthomycin A [40] and albaflavenone [41] are antibiotics or antimicrobial compounds produced by *Streptomyces* GD-4, exhibiting strong bacteriostatic and antifungal properties. Hopene, a terpene compound widely found in bacterial membranes, has a similar secondary metabolite synthesis gene cluster annotated in *Streptomyces collinus* Tü 365 [42]. In addition, ectoine is a natural amino acid derivative that plays a vital role in stress resistance [43]. It provides osmotic protection, exhibits antioxidant properties, and stabilizes protein structures.

Siderophores play a crucial role in chelating iron ions. The synthetic gene cluster responsible for the secondary metabolite amychelin belongs to the type I polyketide synthase (T1PKS) pathway (Table 5). Amychelin is a non-peptide siderophore containing multiple functional groups, such as phenolic hydroxyl and carboxyl groups, that chelate iron to form high-affinity complexes [44]. As a vital secondary metabolite of plant growth-promoting microorganisms, it enhances iron absorption by plant root systems.

## 4. Discussion

Plant growth-promoting rhizobacteria (PGPR) are extensively applied in agricultural production and soil ecological restoration projects [45,46,47]. *Streptomyces*, a prevalent Gram-positive bacterium found in soil, establishes a symbiotic relationship with plants in the rhizosphere [48]. In pot experiments, strain GD-4 significantly enhanced chlorophyll content and root biomass, indicating its potential to promote plant growth in low-fertility soils [49]. Studies have shown that *Streptomyces* species can promote the growth of *Pinus massoniana* seedlings and increase root biomass. Also, in field experiments, the grain yield of wheat supplemented with *Streptomyces* is equal to or even higher than that achieved with nitrogen fertilizer application [50]. Therefore, it is speculated that *Streptomyces* may enhance the ability of plants to absorb and utilize nitrogen from the soil in nitrogen-limited environments, including both nitrate and ammonium forms of nitrogen. In this study, a strain of *Streptomyces* with nitrogen-retention capabilities was isolated from the rhizosphere of a pioneer plant in restored grassland. Inoculation experiments confirmed its ability to colonize the rhizosphere of plants and promote plant growth under oligotrophic soil conditions. To evaluate its potential as a PGPR, whole-genome sequencing using third-generation technology was performed. Genome mining revealed the presence of functional genes associated with both plant growth promotion and abiotic stress alleviation.

For the strain GD-4 genome, primary involvements in nitrogen metabolism and transport, phosphorus metabolism, sulfur metabolism, siderophore production, and auxin synthesis were annotated. Similar genes have also been reported in other plant growth-promoting rhizobacteria (PGPR). Effective colonization of microorganisms in the rhizosphere is fundamental to the functional realization of PGPR [49,51,52,53,54]. Scanning electron microscopy results showed that GD-4 successfully colonized the root systems of three different grasses, and the hyphae and spore structures were visible (Figure 1). Bacterial colonization in plants relies on the recognition of chemical messages and chemotaxis. The GD-4 genome encodes methyl-accepting chemotaxis protein (MCP) and chemotaxis protein methyltransferase (CheR) (Table A6). Methyltransferase (CheR) catalyzes the methylation of the cytoplasmic signaling domain of chemoreceptors and is a core component of the chemosensory cascade [55,56]. Studies have shown that mutations or deletions of CheR can disrupt the chemotactic behavior in various species [56]. Additionally, some research suggests that CheR may play a role in biofilm formation [57]. Meanwhile, according to the gene annotation results, it can use a series of plant-derived compounds as carbon sources for growth (Table A6), transport nutrients such as cellobiose and glucose through ABC transports, and achieve a good symbiotic relationship with plants [58].

**Figure A4:**
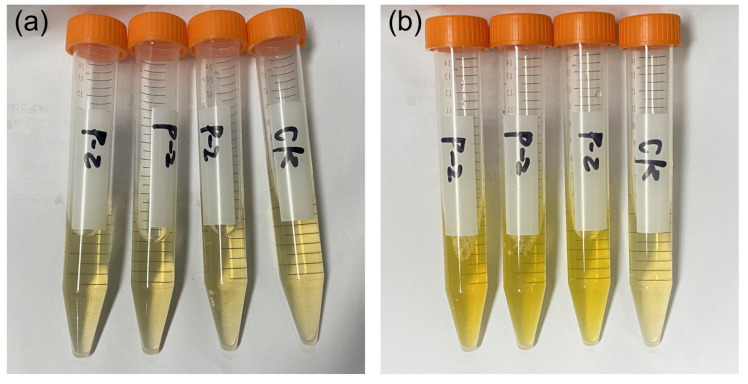
Results of ammonium production experiment: (**a**) before adding Nessler’s reagent (K_2_HgI_4_); (**b**) after adding Nessler’s reagent (K_2_HgI_4_).

It is well established that the most effective method for biological nitrogen fixation is the symbiotic relationship between legumes and rhizobia [59,60,61]. For plants that cannot rely on microbial nitrogen fixation to establish a stable nitrogen source, other nitrogen cycle pathways may exist to provide nitrogen for plant growth [62]. A key characteristic of strain GD-4 is its ability to produce ammonia (Figure A4), a nitrogen source readily available for plant uptake. Ammonia is typically generated through processes such as amino acid degradation, urease-mediated hydrolysis, deamination, and other biological activities. Nitrate, nitrite, and ammonia serve as the primary nitrogen sources in the environment, undergoing transformation and regulation by various environmental factors. Notably, the GD-4 genome predominantly features annotations for the dissimilatory nitrate reduction to ammonium (DNRA) pathway (Figure 6). Current studies on metagenomic and plant growth-promoting bacteria have shown that this pathway plays a role in nitrogen retention in the soil or plant rhizosphere [13,63,64,65]. Several articles have reported that *Pseudomonas* spp. possessing the genes nirB and genes nirD can also perform dissimilatory nitrate reduction to ammonium (DNRA) and convert nitrate nitrogen into ammonium under aerobic conditions [66]. This study predicts that dissimilatory nitrate reduction to ammonium (DNRA) may serve as an effective mechanism for GD-4 to retain nitrogen in this environment [12]. Moreover, studies have shown that the direct assimilation of urine-derived nitrogen into microbial organic nitrogen pools is a crucial process for nitrogen retention in urine patches. This process subsequently supports plant nitrogen supply during microbial turnover [67].

Phosphorus is a crucial nutrient for plants, and its deficiency in available form restricts plant growth and development. The related genes pstA, pstB, and pstC related to phosphorus metabolism were found in the gene annotation of GD-4 and are phosphate transporters, participating in bacterial phosphate transport as phosphate-specific transport (pst) operons. The alkaline phosphatase encoded by the gene phoA can decompose organic phosphorus and release inorganic phosphorus to provide nutritional support for plants [68]. The GD-4 genome encodes multiple genes related to sulfur metabolism and transport (Table A7). Sulfur, as a key element for plant growth, is related to plant stress resistance to a certain extent [69]. For example, the annotated H_2_S synthesis genes can affect plant hormone regulation and participate in plant responses to abiotic stress [32]. Auxin is an essential substance for plant growth, and IAA secretion is one of the important characteristics of some PGPRs [70]. In tryptophan operon, tryptophan synthase alpha subunit (trpA) and beta subunit (trpB) catalyze the conversion of indole to tryptophan, while trpC catalyzes the cyclization reaction to produce indole-3-glycerol phosphate. Additionally, trpD catalyzes the formation of phosphoribosylanthranilate (PRA), and trpE catalyzes the reaction between chorismate and glutamine to generate anthranilate, which serves as a precursor for IAA biosynthesis. *Streptomyces* sp. AC40 has been reported to contain annotated genes encoding nitrile hydratases and to produce IAA via the IAN pathway [71]. There are five tryptophan-dependent IAA biosynthesis pathways in organisms, including the IAM, IAN, indole-3-pyruvic acid (IPyA), tryptamine (TAM), and tryptophan side chain oxidase (TSO) pathways [70,72]. In GD-4, the IAA synthesis pathway integrates multiple pathways, revealing the flexibility of IAA biosynthesis in bacteria (Figure 6).

Another key role of plant growth-promoting bacteria is the production of siderophores [73,74,75]. The GD-4 genome encodes genes involved in siderophore production and transport, including coproporphyrin ferrochelatase, methylglutaconyl-CoA hydratase, shikimate kinase, and ABC transporter permeases. AntiSMASH analysis identified BGCs of the siderophore desferrioxamin B, aerobactin, in the GD-4 genome. Desferrioxamine B and desferrioxamine E are siderophores predicted to be produced by *Streptomyces* sp. GD-4. These compounds primarily support microbial survival and growth in iron-deficient environments by chelating ferric iron (Fe^3+^) and facilitating its transport into cells [76]. Siderophore production by actinomycetes is an important factor in the antagonism of plant pathogens and can produce indirect PGP effects on plants [77,78]. In addition, we have also found many genes responsible for the synthesis of antibacterial compounds. The possible synthesized products include enediyne antibiotics, ansamycins, vancomycin-group antibiotics, and tetracycline. Some secondary metabolites can chelate iron ions, destroy the formation of biofilm, and have good antibacterial effects (Table A8).

Many studies have demonstrated that *Streptomyces* can regulate the structure of soil microbial communities [79]. For instance, Zhang et al.’s study showed that inoculating *Streptomyces* Act12 and D74 into cucumber root soil increased bacterial diversity and recruited more nitrogen-fixing bacteria [80]. Similarly, Hu et al.’s research found that inoculating *Streptomyces TOR3209* into tomato plants not only enhanced the abundance of microorganisms critical to the nitrogen cycle but also facilitated the recruitment of the endophytic growth-promoting bacterium *Bacillus velezensis* WSW007 [81]. Preliminary research in our lab indicates that as grassland degradation deepens, the abundance of *Streptomyces* increases. This suggests that *Streptomyces* species may be particularly well adapted to nutrient-poor soils and have the potential to enhance plant growth by influencing the root microbial community.

To adapt to the abiotic stressors of plateau soils, the genome of *Streptomyces* sp. GD-4 contains genes encoding osmotic regulators, including transporters for trehalose, polyamines, and proline (Table A9). The identification of these genes in the alpine sandy environment provides insight into how GD-4 maintains stable cell morphology under stress. Additionally, the gene cluster responsible for the biosynthesis of glycine betaine further elucidates the bacterium’s capacity to withstand harsh environmental conditions. For example, ectoine, a common compatible solute, enables bacteria to adapt to high-osmotic-pressure environments. Its biosynthesis begins with diaminobutyrate-2-oxoglutarate transaminase (ectB) [82]. This gene cluster (ectABCD), encoding the enzymes for ectoine biosynthesis, was previously identified in the genome of *Streptomyces coelicolor* A3(2) [43]. Moreover, the annotated protein secretion systems in *Streptomyces* sp. GD-4 are primarily the Sec system (post-translational translocation) and the Tat system (twin-arginine translocation), with the core coding genes being secYEG/yajC/yidC and tatABC, respectively [69,83] (Table A10). These secretion systems facilitate the export of adhesion proteins and factors involved in the synthesis of extracellular polysaccharides (EPSs) and auxins, all of which contribute to bacterial colonization and symbiosis with plants [84]. The predicted stress-resistant genes, matching the environmental conditions of isolation, confirm GD-4′s adaptive survival abilities and its potential for promoting plant growth in sandy soil for ecological restoration.

## 5. Conclusions

Our research identified a plant rhizosphere growth-promoting bacterium, GD-4, which promotes the growth of plants in alpine desert grasslands. Additionally, it was confirmed that *Streptomyces* sp. GD-4 exhibits strong colonization capabilities and growth-promoting effects across three species of grasses. This study further elucidates the potential growth-promoting mechanisms of GD-4 through whole-genome and comparative genomics analyses. It is hypothesized that the key mechanism involves its capacity to assist plants in retaining nitrogen in the soil by reducing dissimilatory nitrate reduction to ammonium (DNRA). Nitrate nitrogen is converted into ammonium nitrogen, which is more readily absorbed by plants, thereby indirectly facilitating the initial colonization of plants in desolate grasslands. Additionally, it was found that the bacterium possesses specific stress resistance genes, including those responsible for the production of siderophores, trehalose, and cold- and heat-shock proteins, enabling it to survive in environments characterized by abiotic stress. This suggests that GD-4 is particularly well adapted to the oligotrophic soils of the plateau, thereby promoting plant health and facilitating adaptive growth. These findings indicate that GD-4 has the potential for the development of microbial agents aimed at ecological restoration engineering.

## Figures and Tables

**Table 1:** General features and genomic assembly of *Streptomyces* sp. GD-4.

Features	Chromosome
Total size of the contigs (In Megabases)	9,994,786 bp
Number of protein-coding genes	9352
Number of rRNA genes	18
Number of tRNA genes	71
Number of sRNA genes	62
G + C%	70.46%
Signal transduction	138
Prophage regions	1

**Table 2:** Summary of functional annotations.

Functional Annotations	Number of Protein-Coding Genes (CDs)	Percentage (%)
Total	9352	100
COG	7009	74.9
KEGG	6107	65.3
GO	5435	63.66
NR	9258	58.11
Swiss-port	5902	63.1
Pfam	7426	79.4

**Table 3:** Genes related to nitrogen, phosphorus, and sulfur metabolism.

Function	Gene ID	KO Name	KO ID	KO Description	Enzyme
Nitrogen metabolism	gene2544	gltB	K00265	glutamate synthase (NADPH), large chain	[EC:1.4.1.13]
	gene5942	nirD	K00363	nitrite reductase (NADH), small subunit	[EC:1.7.1.15]
	gene5943	nirB	K00362	nitrite reductase (NADH), large subunit	[EC:1.7.1.15]
	gene4307	narK	K02575	MFS transporter, NNP family, nitrate/nitrite transporter	-
	gene0326	narL	K07684	nitrate/nitrite response regulator NarL, two-component system, NarL family	-
	gene2048	narH	K00371	nitrate reductase/nitrite oxidoreductase, beta subunit	[EC:1.7.5.11.7.99.-]
	gene2049	narJ	K00373	nitrate reductase molybdenum cofactor assembly chaperone NarJ/NarW	-
	gene2544	gltB	K00265	glutamate synthase (NADPH), large chain	[EC:1.4.1.13]
	gene6510	gltD	K00266	glutamate synthase (NADPH), small chain	[EC:1.4.1.13]
	gene1739	glnA	K01915	glutamine synthetase	[EC:6.3.1.2]
	gene2808	glnB	K04751	nitrogen regulatory protein P-II 1	-
	gene2807	glnD	K00990	[protein-PII] uridylyltransferase	[EC:2.7.7.59]
	gene6281	glnE	K00982	[glutamine synthetase] adenylyltransferase/[glutamine synthetase]-adenylyl-L-tyrosine phosphorylase	[EC:2.7.7.42 2.7.7.89]
	gene6662	iscU	K04488	nitrogen fixation protein NifU and related proteins	-
	gene2809	amt	K03320	ammonium transporter, Amt family	-
	gene0085	nasT	K07183	two-component system, response regulator/RNA-binding antiterminator	-
	gene0517	moaC	K03637	cyclic pyranopterin monophosphate synthase	[EC:4.6.1.17]
	gene3219	moaE	K03635	molybdopterin synthase catalytic subunit	[EC:2.8.1.12]
	gene3830	moaA	K03639	GTP 3′,8-cyclase	[EC:4.1.99.22]
	gene4835	moaD	K03636	sulfur-carrier protein	-
	gene8041	urtE	K11963	urea transport system ATP-binding protein	-
	gene8042	urtD	K11962	urea transport system ATP-binding protein	-
	gene8043	urtC	K11961	urea transport system permease protein	-
	gene8044	urtB	K11960	urea transport system permease protein	-
	gene8045	urtA	K11959	urea transport system substrate-binding protein	-
sulfur metabolism	gene1472	cysJ	K00380	sulfite reductase (NADPH) flavoprotein alpha-component	[EC:1.8.1.2]
	gene2334	cysH	K00390	phosphoadenosine phosphosulfate reductase	[EC:1.8.4.8 1.8.4.10]
	gene2335	cysC	K00860	adenylylsulfate kinase	[EC:2.7.1.25]
	gene2336	cysD	K00957	sulfate adenylyltransferase subunit 2	[EC:2.7.7.4]
	gene2337	cysN	K00956	sulfate adenylyltransferase subunit 1	[EC:2.7.7.4]
	gene2339	ssuA	K15553	sulfonate transport system substrate-binding protein	-
	gene2340	ssuB	K15555	sulfonate transport system ATP-binding protein	[EC:7.6.2.14]
	gene2341	ssuC	K15554	sulfonate transport system permease protein	-
	gene5061	ssuD	K04091	alkanesulfonate monooxygenase	[EC:1.14.14.5 1.14.14.34]
	gene2574	sseA	K01011	thiosulfate/3-mercaptopyruvate sulfurtransferase	[EC:2.8.1.1 2.8.1.2]
	gene4389	tauA	K15551	taurine transport system substrate-binding protein	-
	gene4390	tauB	K10831	taurine transport system ATP-binding protein	[EC:7.6.2.7]
	gene4388	tauC	K15552	taurine transport system permease protein	-
	gene4391	tauD	K03119	taurine dioxygenase	[EC:1.14.11.17]
	gene6420	thiS	K03154	sulfur carrier protein	-
Phosphorus metabolism	gene4333	pstC	K02037	phosphate transport system permease protein	-
	gene4334	pstA	K02038	phosphate transport system permease protein	-
	gene4335	pstB	K02036	phosphate transport system ATP-binding protein	[EC:7.3.2.1]
	gene1629	phoD	K01113	alkaline phosphatase D	[EC:3.1.3.1]
	gene6104	phoA	K01077	alkaline phosphatase	[EC:3.1.3.1]
	gene4080	ppx-gppA	K01524	exopolyphosphatase/guanosine-5′-triphosphate,3′-diphosphate pyrophosphatase [EC:3.6.1.11 3.6.1.40]	-
	gene4131	ppa	K01507	inorganic pyrophosphatase	[EC:3.6.1.1]
	gene3124	phoP	K07658	two-component system, OmpR family, alkaline phosphatase synthesis response regulator PhoP	-
	gene3254	phoH	K06217	phosphate starvation-inducible protein PhoH and related proteins	-
	gene4251	phoU	K02039	phosphate transport system protein	-
	gene5771	phoH2	K07175	PhoH-like ATPase	-
	gene0729	gdh	K00034	glucose 1-dehydrogenase	[EC:1.1.1.47]

**Table 4:** Genes and protein products present in the genome of *Streptomyces* sp. GD-4.

Function	Gene ID	KO Name	KO ID	KO Description	Enzyme
Auxin biosynthesis	gene6498	trpA	K01695	tryptophan synthase alpha chain	[EC:4.2.1.20]
	gene6497	trpB	K01696	tryptophan synthase beta chain	[EC:4.2.1.20]
	gene6496	trpC	K01609	indole-3-glycerol phosphate synthase	[EC:4.1.1.48]
	gene6381	trpD	K00766	anthranilate phosphoribosyltransferase	[EC:2.4.2.18]
	gene6492	trpE	K01657	anthranilate synthase component I	[EC:4.1.3.27]
	gene5439	nthB	K20807	nitrile hydratase subunit beta	[EC:4.2.1.84]
	gene5441	nthA	K01721	nitrile hydratase subunit alpha	[EC:4.2.1.84]
	gene0620	-	K00128	aldehyde dehydrogenase (NAD+)	[EC:1.2.1.3]
	gene1290	-	K01593	aromatic-L-amino-acid/L-tryptophan decarboxylase	[EC:4.1.1.28 4.1.1.105]
	gene9104	-	K01501	nitrilase	[EC:3.5.5.1]
	gene4607	hisC	K00817	histidinol-phosphate aminotransferase	[EC:2.6.1.9]
Abiotic stress tolerance	gene2632	ectB	K00836	diaminobutyrate-2-oxoglutarate transaminase	[EC:2.6.1.76]
	gene6739	ectD	K10674	ectoine hydroxylase	[EC:1.14.11.55]
	gene6740	ectC	K06720	L-ectoine synthase	[EC:4.2.1.108]
	gene6742	ectA	K06718	L-2,4-diaminobutyric acid acetyltransferase	[EC:2.3.1.178]
	gene3683	groEL	K04077	chaperonin GroEL	[EC:5.6.1.7]
	gene3684	groES	K04078	chaperonin GroES	-
	gene3608	betB	K00130	betaine-aldehyde dehydrogenase [EC:1.2.1.8]	-
	gene1092	betA	K00108	choline dehydrogenase [EC:1.1.99.1]	-
	gene1761	betI	K02167	TetR/AcrR family transcriptional regulator, transcriptional repressor of bet genes	
	gene5096	cspA	K03704	cold-shock protein	-
	gene4328	hspR	K13640	MerR family transcriptional regulator, heat-shock protein HspR	-
	gene4731	hslJ	K03668	heat-shock protein HslJ	-
	gene3770	htpX	K03799	heat-shock protein HtpX	[EC:3.4.24.-]
	gene6571	hslR	K04762	ribosome-associated heat-shock protein Hsp15	-
	gene5866	hrcA	K03705	heat-inducible transcriptional repressor	-
	gene1087	proW	K02001	glycine betaine/proline transport system permease protein	-
	gene1088	proV	K02000	glycine betaine/proline transport system ATP-binding protein	[EC:7.6.2.9]
	gene1866	proP	K03762	MFS transporter, MHS family, proline/betaine transporter	-
	gene2716	proS	K01881	prolyl-tRNA synthetase	[EC:6.1.1.15]
	gene4311	proA	K00147	glutamate-5-semialdehyde dehydrogenase	[EC:1.2.1.41]
	gene5828	proB	K00931	glutamate 5-kinase	[EC:2.7.2.11]
	gene2163	ggt	K00681	gamma-glutamyltranspeptidase/glutathione hydrolase	[EC:2.3.2.2 3.4.19.13]
	gene0962	gst	K00799	glutathione S-transferase	[EC:2.5.1.18]
	gene7747	trxA	K03671	thioredoxin 1	-
	gene4564	trxB	K00384	thioredoxin reductase (NADPH)	[EC:1.8.1.9]
	gene2356	treX	K01214	isoamylase	[EC:3.2.1.68]
	gene2358	treY	K06044	(1->4)-alpha-D-glucan 1-alpha-D-glucosylmutase	[EC:5.4.99.15]
	gene2985	treS	K05343	maltose alpha-D-glucosyltransferase/alpha-amylase	[EC:5.4.99.16 3.2.1.1]
	gene4925	otsB	K01087	trehalose 6-phosphate phosphatase	[EC:3.1.3.12]
	gene4926	otsA	K00697	trehalose 6-phosphate synthase	[EC:2.4.1.15 2.4.1.347]
Iron uptake and transport	gene8395	fhuB	K23228	ferric hydroxamate transport system permease protein	-
	gene8396	fhuD	K23227	ferric hydroxamate transport system substrate-binding protein	-
	gene8397	fhuC	K10829	ferric hydroxamate transport system ATP-binding protein	[EC:7.2.2.16]
	gene3465	fepD	K23186	iron-siderophore transport system permease protein	-
	gene3466	fepG	K23187	iron-siderophore transport system permease protein	-
	gene6856	fepC	K23188	iron-siderophore transport system ATP-binding protein	[EC:7.2.2.177.2.2.-]
	gene5573	desE	K25287	iron-desferrioxamine transport system substrate-binding protein	-
	gene5604	entS	K08225	MFS transporter, ENTS family, enterobactin (siderophore) exporter	-

**Table 5:** Predicted secondary metabolite biosynthetic gene clusters in *Streptomyces* sp. GD-4.

Cluster ID	Type	Similar Cluster	Similarity (%)	Gene No.
cluster1	butyrolactone	salinomycin	4	11
cluster2	butyrolactone	merochlorin A/merochlorin B	78	118
cluster1	NRPS	griseochelin	100	85
cluster2	RiPP-like	informatipeptin	37	6
cluster3	terpene	hopene	92	22
cluster4	siderophore	grincamycin	8	13
cluster5	NRPS-like	s56-p1	11	35
cluster6	terpene	geosmin	100	18
cluster7	RiPP-like	-	-	12
cluster8	siderophore	-	-	7
cluster9	terpene	albaflavenone	100	21
cluster10	terpene	naphthomycin A	9	22
cluster11	NRPS-like	A40926	7	41
cluster12	siderophore	desferrioxamin B/desferrioxamine E	83	9
cluster13	melanin	istamycin	5	11
cluster14	NRPS-like	cyphomycin	2	35
cluster15	ectoine	ectoine	100	10
cluster16	NAPAA	-	-	32
cluster17	NRPS	herboxidiene	4	36
cluster18	T1PKS	amychelin	81	74
cluster19	melanin	melanin	71	9
cluster20	T1PKS	spore pigment	83	130
cluster21	terpene	2-methylisoborneol	100	17
cluster22	NRPS	lasalocid	9	75
cluster23	NRPS	tylactone	6	21
cluster24	RRE-containing	mycotrienin I	7	30
cluster25	terpene	-	-	21

## Data Availability

Dataset available on request from the authors.

## Appendix A

**Table A1:** Features of the GD-4 genome in comparison with other *Streptomyces* strains from different ecological niches.

Name	Source of Isolation	Genome Size	CD’s	G + C (%)	Potential Function	Accession No.	Reference
*Streptomyces rochei* S32	soil	8.0 Mb	7486	72.5	Plant growth promotion, nitrogen fixation, production of bioactive substances	CP133098	[85]
*Streptomyces griseoincarnatus* HNS054	Marine sponge	7.5 Mb	6678	72.3	Heterologous expression host for secondary metabolites, salinity tolerance	CP139576	[86]
*Streptomyces* sp. AFD10	desert soil	7.9 Mb	7246	72.6	Antibiotic production, bioactive secondary metabolite production	JASSVZ000000000	[87]
*Streptomyces* sp. Z38	root tissues	7.3 Mb	6446	72.4	Plant growth promotion, heavy metal resistance, bio-nanoparticle synthesis	WPIR00000000	[88]
*Streptomyces luteireticuli* ASG80	Sisal roots	8.7 Mb	7867	71.77	Biocontrol agent against Phytophthora diseases	CP167927	[89]
*Streptomyces* sp. UYFA156	seeds	7.1 Mb	-	73.4	Plant growth promotion	CP040466	[90]
*Streptomyces* sp. C8S0	sediment	6.9 Mb	6,841	73.5	Bioactive metabolite production	CP045031	[91]
*Streptomyces* sp. JL1001	Rhizosphere soil	7.9 Mb	7315	71.71	Production of novel bioactive natural products, leinamycin-type gene cluster	CP136798	[92]
*Streptomyces* sp.CH9-7^T^	Rhizosphere soil	8.8 Mb	7664	71.7	Nocardamine production, bioactive secondary metabolite production	JAERRI000000000	[93]
*Streptomyces*_sp_HP-A2021	Rhizosphere soil	9.6 Mb	8534	71.07	Bioactive secondary metabolite production, antimicrobial activity	CP094344.1	[94]

**Table A2:** Genes involved in nitrogen metabolism predicted for *Streptomyces* sp. GD-4.

Activity Description	Gene ID	KO Name	KO ID	KO Description	Enzyme
Nitrogen metabolism	gene0092	cynT	K01673	carbonic anhydrase	[EC:4.2.1.1]
	gene1739	glnA	K01915	glutamine synthetase	[EC:6.3.1.2]
	gene1760	gdhA	K00261	glutamate dehydrogenase (NAD(P)+)	[EC:6.3.1.2]
	gene2048	narH	K00371	nitrate reductase/nitrite oxidoreductase, beta subunit	[EC:1.7.5.1 1.7.99.-]
	gene2544	gltB	K00265	glutamate synthase (NADPH) large chain	[EC:1.4.1.13]
	gene4307	narK	K02575	MFS transporter, NNP family, nitrate/nitrite transporter	-
	gene4308	nasC	K00372	assimilatory nitrate reductase catalytic subunit	[EC:1.7.99.-]
	gene4309	nasB	K00360	assimilatory nitrate reductase electron transfer subunit	[EC:1.7.99.-]
	gene5295	nirK	K00368	nitrite reductase (NO-forming)	[EC:1.7.2.1]
	gene5356	-	K15371	glutamate dehydrogenase	[EC:1.4.1.2]
	gene5868	npd	K00459	nitronate monooxygenase	[EC:1.13.12.16]
	gene5942	nirD	K00363	nitrite reductase (NADH), small subunit	[EC:1.7.1.15]
	gene5943	nirB	K00362	nitrite reductase (NADH), large subunit	[EC:1.7.1.15]
	gene6510	gltD	K00266	glutamate synthase (NADPH), small chain	[EC:1.4.1.13]
	gene9104	-	K01501	nitrilase	[EC:3.5.5.1]
	gene6662	iscU	K04488	nitrogen fixation protein NifU and related proteins	-
	gene0326	narL	K07684	two-component system, NarL family, nitrate/nitrite response regulator NarL	-
	gene2048	narH	K00371	nitrate reductase/nitrite oxidoreductase, beta subunit	[EC:1.7.5.1/1.7.99.-]
	gene2049	narJ	K00373	nitrate reductase molybdenum cofactor assembly chaperone NarJ/NarW	-
	gene4268	narA	K25150	polyether ionophore transport system ATP-binding protein	-
	gene4269	narB	K25149	polyether ionophore transport system permease protein	-
Molybdenum cofactor	gene4499	mobA	K03752	molybdenum cofactor guanylyltransferase	[EC:2.7.7.77]
	gene4379	moeB	K21029	molybdopterin-synthase adenylyltransferase	[EC:2.7.7.80]
	gene0517	moaC	K03637	cyclic pyranopterin monophosphate synthase	[EC:4.6.1.17]
	gene3219	moaE	K03635	molybdopterin synthase catalytic subunit	[EC:2.8.1.12]
	gene3830	moaA	K03639	GTP 3′,8-cyclase	[EC:4.1.99.22]
	gene4835	moaD	K03636	sulfur-carrier protein	-
Nitrogen transporters	gene0085	nasT	K07183	two-component system, response regulator/RNA-binding antiterminator	-
	gene2809	amt	K03320	ammonium transporter, Amt family	-
	gene1739	glnA	K01915	glutamine synthetase	[EC:6.3.1.2]
	gene2807	glnD	K00990	[protein-PII] uridylyltransferase	[EC:2.7.7.59]
	gene2808	glnB	K04751	nitrogen regulatory protein P-II 1	-
	gene6281	glnE	K00982	[glutamine synthetase] adenylyltransferase/[glutamine synthetase]-adenylyl-L-tyrosine phosphorylase	[EC:2.7.7.42/2.7.7.89]
	gene4307	narK	K02575	MFS transporter, NNP family, nitrate/nitrite transporter	-

**Table A3:** Genes involved in phosphate solubilization predicted for Streptomyces sp. GD-4.

Activity Description	Gene ID	KO Name	KO ID	KO Description	Enzyme
Phosphate solubilization	gene1931	zwf	K00036	glucose-6-phosphate 1-dehydrogenase	[EC:1.1.1.49/1.1.1.363]
	gene4131	ppa	K01507	inorganic pyrophosphatase	[EC:3.6.1.1]
	gene0144	pqqE	K06139	PqqA peptide cyclase	[EC:1.21.98.4]
	gene2585	pqqL	K07263	zinc protease	[EC:3.4.24.-]
	gene0729	gdh	K00034	glucose 1-dehydrogenase	[EC:1.1.1.47]
	gene1760	gdhA	K00261	glutamate dehydrogenase(NAD(P)+)	[EC:1.4.1.3]
	gene0424	phoD	K01113	alkaline phosphatase D	[EC:3.1.3.1]
	gene8518	phoP	K07658	two-component system, OmpR family, alkaline phosphatase synthesis response regulator PhoP	-
	gene3368	phoH2	K07175	PhoH-like ATPase	-
	gene4887	phoU	K02039	phosphate transport system protein	-
	gene5884	phoH	K06217	phosphate starvation-inducible protein PhoH and related proteins	-
	gene6104	phoA	K01077	alkaline phosphatase	[EC:3.1.3.1]
Phosphate transport	gene4252	pstS	K02040	phosphate transport systemsubstrate-binding protein	-
	gene4803	pstB	K02036	phosphate transport systemATP-binding protein	[EC:7.3.2.1]
	gene4804	pstA	K02038	phosphate transport systempermease protein	-
	gene4805	pstC	K02037	phosphate transport systempermease protein	-
	gene6940	pstI	K20754	aqualysin 1	[EC:3.4.21.111]
	gene1103	phnB	K04750	PhnB protein	-
	gene1789	phnW	K03430	2-aminoethylphosphonate-pyruvate transaminase	[EC:2.6.1.37]
	gene4587	phnO	K09994	(aminoalkyl)phosphonate N-acetyltransferase	[EC:2.3.1.280]
	gene7793	phnA	K19670	phosphonoacetate hydrolase	[EC:3.11.1.2]

**Table A4:** Genes involved in auxin metabolism predicted for *Streptomyces* sp. GD-4.

Activity Description	Gene ID	KO Name	KO ID	KO Description	Enzyme
IAA biosynthesis	gene0221	paaF	K01692	enoyl-CoA hydratase	[EC:4.2.1.17]
	gene0256	amiE	K01426	amidase	[EC:3.5.1.4]
	gene0482	gcdH	K00252	glutaryl-CoA dehydrogenase	[EC:1.3.8.6]
	gene0485	atoB	K00626	acetyl-CoA C-acetyltransferase	[EC:2.3.1.9]
	gene0620	-	K00128	aldehyde dehydrogenase (NAD+)	[EC:1.2.1.3]
	gene1290	-	K01593	aromatic-L-amino-acid/L-tryptophan decarboxylase	[EC:4.1.1.28/4.1.1.105]
	gene1432	cypD_E	K14338	cytochrome P450/NADPH-cytochrome P450 reductase	[EC:1.14.14.1/1.6.2.4]
	gene1474	pdhD	K00382	dihydrolipoyl dehydrogenase	[EC:1.8.1.4]
	gene1502	-	K03392	aminocarboxymuconate-semialdehyde decarboxylase	[EC:4.1.1.45]
	gene1818	fadJ	K01782	3-hydroxyacyl-CoA dehydrogenase/enoyl-CoA hydratase/3-hydroxybutyryl-CoA epimerase	[EC:1.1.1.35/4.2.1.17/5.1.2.3]
	gene2069	katE	K03781	catalase	[EC:1.11.1.6]
	gene2370	phsA	K20219	o-aminophenol oxidase	[EC:1.10.3.4]
	gene2538	katG	K03782	catalase-peroxidase	[EC:1.11.1.21]
	gene4279	kynU	K01556	kynureninase	[EC:3.7.1.3]
	gene4282	kynA	K00453	tryptophan 2,3-dioxygenase	[EC:1.13.11.11]
	gene4301	aofH	K00274	monoamine oxidase	[EC:1.4.3.4]
	gene5439	nthB	K20807	nitrile hydratase subunit beta	[EC:4.2.1.84]
	gene5441	nthA	K01721	nitrile hydratase subunit alpha	[EC:4.2.1.84]
	gene7158	-	K22450	aralkylamine N-acetyltransferase	[EC:2.3.1.87]
	gene9104	-	K01501	nitrilase	[EC:3.5.5.1]
	gene6498	trpA	K01695	tryptophan synthase alpha chain	[EC:4.2.1.20]
	gene6497	trpB	K01696	tryptophan synthase beta chain	[EC:4.2.1.20]
	gene6496	trpC	K01609	indole-3-glycerol phosphate synthase	[EC:4.1.1.48]
	gene6381	trpD	K00766	anthranilate phosphoribosyltransferase	[EC:2.4.2.18]
	gene6492	trpE	K01657	anthranilate synthase component I	[EC:4.1.3.27]
	gene4607	hisC	K00817	histidinol-phosphate aminotransferase	[EC:2.6.1.9]
ACC Deaminase	gene2178	map	K01265	methionyl aminopeptidase	[EC:3.4.11.18]
	gene1750	kbl	K00639	glycine C-acetyltransferase	[EC:2.3.1.29]
	gene1790	pgsA	K00995	CDP-diacylglycerol---glycerol-3-phosphate 3-phosphatidyltransferase	[EC:2.7.8.5]
	gene2753	glpQ	K01126	glycerophosphoryl diester phosphodiesterase	[EC:3.1.4.46]

**Table A5:** Genes involved in antibiotics predicted for Streptomyces sp. GD-4.

Activity Description	Gene ID	KO Name	KO ID	KO Description	Enzyme
Enediyne antibiotics	gene1384	ncsB3	K20420	2-hydroxy-5-methyl-1-naphthoate 7-hydroxylase	[EC:1.14.15.31]
	gene2301	mdpB3	K21193	acetyltransferase/esterase	-
	gene2929	ncsB4	K21209	acyltransferase	-
	gene3244	ncsC4	K21214	NDP-hexose 4-ketoreductase	-
	gene4917	sgcE6	K21185	flavin reductase	-
	gene4990	sgcE11	K21167	enediyne biosynthesis protein E11	-
	gene6898	sgcF	K21159	epoxide hydrolase	-
	gene7800	cepH	K16431	FAD-dependent halogenase	[EC:1.14.19.-]
	gene8258	sgcD3	K21177	cytochrome P450 hydroxylase	[EC:1.14.-.-]
	gene8273	calE5	K21172	enediyne biosynthesis protein CalE5	-
Ansamycins	gene1929	tktA	K00615	transketolase	[EC:2.2.1.1]
	gene8433	asm10	K16038	N-methyltransferase	[EC:2.1.1.-]
Vancomycin-group antibiotics	gene0345	evaC	K16437	methylation protein EvaC	-
	gene1329	rfbB	K01710	dTDP-glucose 4,6-dehydratase	[EC:4.2.1.46]
	gene7800	cepH	K16431	FAD-dependent halogenase	[EC:1.14.19.-]
	gene7828	cepJ	K16434	thioesterase CepJ	-
	gene8312	nocN	K16422	4-hydroxymandelate oxidase	[EC:1.1.3.46]
	pA_gene0134	cepA	K16428	nonribosomal peptide synthetase CepA	-
Tetracycline	gene1025	tetX	K18221	tetracycline 11a-monooxygenase, tetracycline resistance protein	[EC:1.14.13.231]
	gene3288	oxyQ	K14254	aminotransferase	-
	gene5733	oxyS	K14256	anhydrotetracycline 6-monooxygenase/5a,11a-dehydrotetracycline 5-monooxygenase	[EC:1.14.13.38/1.14.13.234]
	gene8337	oxyF	K14251	C-methyltransferase	[EC:2.1.1.-]
	pA_gene0186	oxyA	K05551	minimal PKS ketosynthase (KS/KS alpha)	[EC:2.3.1.-/2.3.1.260/2.3.1.235]
	pA_gene0187	oxyB	K05552	minimal PKS chain-length factor (CLF/KS beta)	[EC:2.3.1.-2.3.1.260 2.3.1.235]
	pA_gene0188	ctcP	K14257	tetracycline 7-halogenase/FADH2 O2-dependent halogenase	[EC:1.14.19.49/1.14.19.-]
	pA_gene0199	oxyC	K05553	minimal PKS acyl carrier protein	-
	pA_gene0200	oxyJ	K12420	ketoreductase	[EC:1.1.1.-]
Phenazine	gene2251	phzS	K20940	5-methylphenazine-1-carboxylate 1-monooxygenase	[EC:1.14.13.218]
	gene6250	phzF	K06998	trans-2,3-dihydro-3-hydroxyanthranilate isomerase	[EC:5.3.3.17]
	gene7406	phzF	K06998	trans-2,3-dihydro-3-hydroxyanthranilate isomerase	[EC:5.3.3.17]
	gene6410	phzE	K13063	2-amino-4-deoxychorismate synthase	[EC:2.6.1.86]

**Table A6:** Genes involved in plant–bacteria interactions predicted for *Streptomyces* sp. GD-4.

Activity Description	Gene ID	KO Name	KO ID	KO Description	Enzyme
Root colonization Motility	gene8106	flgS	K02482	two-component system, NtrC family, sensor kinase	[EC:2.7.13.3]
	gene2789	fliA	K02405	RNA polymerase sigma factor FliA	-
	gene2607	rpoD	K03086	RNA polymerase primary sigma factor	-
Root colonization and interactions	gene3612	mdh	K00024	malate dehydrogenase	[EC:1.1.1.37]
	gene0159	xerD	K04763	integrase/recombinase XerD	-
Chemotaxis	gene0567	-	K11354	two-component system, chemotaxis family, sensor kinase Cph1	[EC:2.7.13.3]
	gene0888	rbsB	K10439	ribose transport system substrate-binding protein	-
	gene8627	mcp	K03406	methyl-accepting chemotaxis protein	-
	gene3596	cheR	K00575	chemotaxis protein methyltransferase CheR	[EC:2.1.1.80]
Cellulose degradation	gene0446	bglX	K05349	beta-glucosidase	[EC:3.2.1.21]
	gene0445	-	K01179	endoglucanase	[EC:3.2.1.4]
	gene0447	cbhA	K19668	cellulose 1,4-beta-cellobiosidase	[EC:3.2.1.91]
	gene1904	celF	K01222	6-phospho-beta-glucosidase	[EC:3.2.1.86]
Exopolysaccharide biosynthesis	gene1005	exoZ	K16568	exopolysaccharide production protein ExoZ	-
	gene1536	exoY	K16566	exopolysaccharide production protein ExoY	-
	gene3072	wecA	K02851	UDP-GlcNAc:undecaprenyl-phosphate/decaprenyl-phosphate GlcNAc-1-phosphate transferase	[EC:2.7.8.33/2.7.8.35]
	gene5303	exoA	K16557	succinoglycan biosynthesis protein ExoA	[EC:2.4.-.-]
	gene5659	gumD	K13656	undecaprenyl-phosphate glucose phosphotransferase	[EC:2.7.8.31]
	gene5724	cysE	K00640	serine O-acetyltransferase	[EC:2.3.1.30]
Biofilm formation	gene1169	oxyR	K04761	LysR family transcriptional regulator, hydrogen peroxide-inducible gene activator	-
	gene1609	glgC	K00975	glucose-1-phosphate adenylyltransferase	[EC:2.7.7.27]
	gene2789	fliA	K02405	RNA polymerase sigma factor FliA	-
	gene2982	glgP	K00688	glycogen phosphorylase	[EC:2.4.1.1]
	gene3721	bcsA	K00694	cellulose synthase (UDP-forming)	[EC:2.4.1.12]
	gene3765	crp	K10914	CRP/FNR family transcriptional regulator, cyclic AMP receptor protein	-
	gene4594	gcvA	K03566	LysR family transcriptional regulator, glycine cleavage system transcriptional activator	-
	gene5656	pgaB	K11931	poly-beta-1,6-N-acetyl-D-glucosamine N-deacetylase	[EC:3.5.1.-]
	gene6457	dksA	K06204	RNA polymerase-binding transcription factor	
	gene7372	crr	K02777	sugar PTS system EIIA component	[EC:2.7.1.-]
	gene8276	rcdA	K23778	TetR/AcrR family transcriptional regulator, regulator of biofilm formation and stress response	-
	gene4092	icaR	K21453	TetR/AcrR family transcriptional regulator, biofilm operon repressor	-

**Table A7:** Genes involved in sulfur metabolism predicted for *Streptomyces* sp. GD-4.

Activity Description	Gene ID	KO Name	KO ID	KO Description	Enzyme
Sulfur metabolism	gene0895	sfnG	K17228	dimethylsulfone monooxygenase	[EC:1.14.14.35]
	gene0932	-	K00387	sulfite oxidase	[EC:1.8.3.1]
	gene1472	cysJ	K00380	sulfite reductase (NADPH) flavoprotein alpha-component	[EC:1.8.1.2]
	gene2332	sir	K00392	sulfite reductase (ferredoxin)	[EC:1.8.7.1]
	gene2334	cysH	K00390	phosphoadenosine phosphosulfate reductase	[EC:1.8.4.8/1.8.4.10]
	gene2335	cysC	K00860	adenylylsulfate kinase	[EC:2.7.1.25]
	gene2336	cysD	K00957	sulfate adenylyltransferase subunit 2	[EC:2.7.7.4]
	gene2337	cysN	K00956	sulfate adenylyltransferase subunit 1	[EC:2.7.7.4]
	gene2574	sseA	K01011	thiosulfate/3-mercaptopyruvate sulfurtransferase	[EC:2.8.1.1/2.8.1.2]
	gene3444	metB	K01739	cystathionine gamma-synthase	[EC:2.5.1.48]
	gene3652	doxD	K16937	thiosulfate dehydrogenase (quinone) large subunit	[EC:1.8.5.2]
	gene4896	doxD	K16937	thiosulfate dehydrogenase (quinone) large subunit	[EC:1.8.5.2]
	gene3878	aprA	K00394	adenylylsulfate reductase, subunit A	[EC:1.8.99.2]
	gene4304	metX	K00641	homoserine O-acetyltransferase/O-succinyltransferase	[EC:2.3.1.31/2.3.1.46]
	gene4469	dmdC	K20035	3-(methylsulfanyl)propanoyl-CoA dehydrogenase	[EC:1.3.99.41]
	gene4657	dmdB	K20034	3-(methylthio)propionyl---CoA ligase	[EC:6.2.1.44]
	gene5061	ssuD	K04091	alkanesulfonate monooxygenase	[EC:1.14.14.5/ 1.14.14.34]
	gene5724	cysE	K00640	serine O-acetyltransferase	[EC:2.3.1.30]
	gene5725	cysK	K01738	cysteine synthase	[EC:2.5.1.47]
	gene6420	thiS	K03154	sulfur carrier protein	-
	gene6037	iscR	K13643	Rrf2 family transcriptional regulator, iron–sulfur cluster assembly transcription factor	-
	gene6382	sufS	K11717	cysteine desulfurase/selenocysteine lyase	[EC:2.8.1.7/4.4.1.16]
	gene6657	sufB	K09014	Fe-S cluster assembly protein SufB	-
	gene6658	sufD	K09015	Fe-S cluster assembly protein SufD	-
	gene6660	sufC	K09013	Fe-S cluster assembly ATP-binding protein	-
Sulfur transport	gene2339	ssuA	K15553	sulfonate transport system substrate-binding protein	-
	gene2340	ssuB	K15555	sulfonate transport system ATP-binding protein	[EC:7.6.2.14]
	gene2341	ssuC	K15554	sulfonate transport system permease protein	-
	gene4388	tauC	K15552	taurine transport system permease protein	-
	gene4389	tauA	K15551	taurine transport system substrate-binding protein	-
	gene4390	tauB	K10831	taurine transport system ATP-binding protein	[EC:7.6.2.7]
	gene4391	tauD	K03119	taurine dioxygenase	[EC:1.14.11.17]

**Table A8:** Genes involved in siderophore biosynthesis predicted for *Streptomyces* sp. GD-4.

Activity Description	Gene ID	KO Name	KO ID	KO Description	Enzyme
Siderophore biosynthesis	gene7262	aroC	K01736	chorismate synthase	[EC:4.2.3.5]
	gene6884	aroH	K06208	chorismate mutase	[EC:5.4.99.5]
	gene2570	hemH	K01772	protoporphyrin/coproporphyrin ferrochelatase	[EC:4.98.1.14.99.1.9]
	gene7265	aroK	K00891	shikimate kinase	[EC:2.7.1.71]
	gene6413	bfr	K03594	bacterioferritin	[EC:1.16.3.1]
	gene6412	bfd	K02192	bacterioferritin-associated ferredoxin	-
	gene3085	lysA	K01586	diaminopimelate decarboxylase	[EC:4.1.1.20]
	gene6037	iscR	K13643	Rrf2 family transcriptional regulator, iron–sulfur cluster assembly transcription factor	-
	gene2932	iscS	K04487	cysteine desulfurase	[EC:2.8.1.7]
	gene6243	efeU	K07243	high-affinity iron transporter	-
	gene7987	pvdQ	K07116	acyl-homoserine-lactone acylase	[EC:3.5.1.97]
	gene0300	mbtH	K05375	MbtH protein	-
	gene4994	mbtN	K00257	acyl-ACP dehydrogenase	[EC:1.3.99.-]
	gene7826	mbtI	K04781	salicylate synthetase	[EC:5.4.4.2/4.2.99.21]
	gene7834	mbtB	K04788	mycobactin phenyloxazoline synthetase	-
	gene2630	iucB	K03896	acetyl CoA:N6-hydroxylysine acetyl transferase	[EC:2.3.1.102]
	gene5568	iucC	K03895	aerobactin synthase	[EC:6.3.2.39]
	gene5570	iucD	K03897	lysine N6-hydroxylase	[EC:1.14.13.59]
	gene0303	dhbF	K04780	glyine—[glycyl-carrierprotein]ligase	[EC:6.2.1.66]
	gene2631	asbA	K24108	spermidine-citrateligase	[EC:6.3.2.-]
	gene5422	entF	K02364	L-serine—[L-seryl-carrierprotein]ligase	[EC:6.3.2.1/46.2.1.72]
	gene7825	entE	K02363	2,3-dihydroxybenzoate—[aryl-carrierprotein]ligase	[EC:6.3.2.1/46.2.1.71]
	gene8325	menF	K02552	menaquinone-specificisochorismatesynthase	[EC:5.4.4.2]
Iron uptake and transport	gene8395	fhuB	K23228	ferric hydroxamate transport system permease protein	-
	gene8396	fhuD	K23227	ferric hydroxamate transport system substrate-binding protein	-
	gene8397	fhuC	K10829	ferric hydroxamate transport system ATP-binding protein	[EC:7.2.2.16]
	gene3465	fepD	K23186	iron-siderophore transport system permease protein	-
	gene3466	fepG	K23187	iron-siderophore transport system permease protein	-
	gene6856	fepC	K23188	iron-siderophore transport system ATP-binding protein	[EC:7.2.2.1/77.2.2.-]
	gene5573	desE	K25287	iron-desferrioxamine transport system substrate-binding protein	-
	gene5604	entS	K08225	MFS transporter, ENTS family, enterobactin (siderophore) exporter	-
	gene6244	efeB	K16301	deferrochelatase/peroxidase EfeB	[EC:1.11.1.-]
	gene6245	efeO	K07224	iron uptake system component EfeO	-
	gene7837	pvdA	K10531	L-ornithine N5-monooxygenase	[EC:1.14.13.1951.14.13.196]

**Table A9:** Genes involved in abiotic stress response predicted for *Streptomyces* sp. GD-4.

Activity Description	Gene ID	KO Name	KO ID	KO Description	Enzyme
Heat tolerance	gene4731	hslJ	K03668	heat-shock protein HslJ	-
	gene3770	htpX	K03799	heat-shock protein HtpX	[EC:3.4.24.-]
	gene6571	hslR	K04762	ribosome-associated heat-shock protein Hsp15	-
	gene5866	hrcA	K03705	heat-inducible transcriptional repressor	-
Cold-shock protein	gene4329	dnaJ	K03686	molecular chaperone DnaJ	-
	gene0076	dnaK	K04043	molecular chaperone DnaK	-
	gene0819	cspA	K03704	cold-shock protein	-
	gene3683	groEL	K04077	chaperonin GroEL	[EC:5.6.1.7]
	gene3684	groES	K04078	chaperonin GroES	-
Salinity tolerance	gene1089	betB	K00130	betaine-aldehyde dehydrogenase	[EC:1.2.1.8]
	gene1092	betA	K00108	choline dehydrogenase	[EC:1.1.99.1]
	gene1761	betI	K02167	TetR/AcrR family transcriptional regulator, transcriptional repressor of bet genes	-
	gene1086	proX	K02002	glycine betaine/proline transport system substrate-binding protein	-
	gene1087	proW	K02001	glycine betaine/proline transport system permease protein	-
	gene1088	proV	K02000	glycine betaine/proline transport system ATP-binding protein	[EC:7.6.2.9]
	gene1866	proP	K03762	MFS transporter, MHS family, proline/betaine transporter	-
	gene2716	proS	K01881	prolyl-tRNA synthetase	[EC:6.1.1.15]
	gene4068	proC	K00286	pyrroline-5-carboxylate reductase	[EC:1.5.1.2]
	gene4311	proA	K00147	glutamate-5-semialdehyde dehydrogenase	[EC:1.2.1.41]
	gene5828	proB	K00931	glutamate 5-kinase	[EC:2.7.2.11]
	gene2892	putR	K23253	PucR family transcriptional regulator, proline-responsive transcriptional activator	-
Oxidative stress tolerance	gene2069	katE	K03781	catalase	[EC:1.11.1.6]
	gene2538	katG	K03782	catalase-peroxidase	[EC:1.11.1.21]
	gene2163	ggt	K00681	gamma-glutamyltranspeptidase/glutathione hydrolase	[EC:2.3.2.2] [EC:3.4.19.13]
	gene0778	trxA	K03671	thioredoxin 1	-
	gene4564	trxB	K00384	thioredoxin reductase (NADPH)	[EC:1.8.1.9]
Polyamine biosynthesis	gene4295	speE	K00797	spermidine synthase	[EC:2.5.1.16]
	gene4902	speG	K00657	diamine N-acetyltransferase	[EC:2.3.1.57]
	gene8127	speA	K01585	arginine decarboxylase	[EC:4.1.1.19]
	gene0359	argS	K01887	arginyl-tRNA synthetase	[EC:6.1.1.19]
	gene7176	argH	K01755	argininosuccinate lyase	[EC:4.3.2.1]
	gene7525	argO	K22477	N-acetylglutamate synthase	[EC:2.3.1.1]
	gene2745	potC	K11070	spermidine/putrescine transport system permease protein	-
	gene2746	potB	K11071	spermidine/putrescine transport system permease protein	-
	gene2747	potA	K11072	spermidine/putrescine transport system ATP-binding protein	[EC:7.6.2.11]
	gene2748	potD	K11069	spermidine/putrescine transport system substrate-binding protein	-
Trehalose	gene2358	treY	K06044	(1->4)-alpha-D-glucan 1-alpha-D-glucosylmutase	[EC:5.4.99.15]
	gene2363	treZ	K01236	maltooligosyltrehalose trehalohydrolase	[EC:3.2.1.141]
	gene2985	treS	K05343	maltose alpha-D-glucosyltransferase/alpha-amylase	[EC:5.4.99.16] [EC:3.2.1.1]
	gene1426	otsB	K01087	trehalose 6-phosphate phosphatase	[EC:3.1.3.12]
	gene4926	otsA	K00697	trehalose 6-phosphate synthase	[EC:2.4.1.15/2.4.1.347]
Tolerance against metal toxicity	gene1479	chrR	K19784	chromate reductase, NAD(P)H dehydrogenase (quinone)	-
	gene8824	chrA	K07240	chromate transporter	-
	gene0716	cusR	K07665	two-component system, OmpR family, copper resistance phosphate regulon response regulator CusR	-
	gene5624	copA	K17686	P-type Cu+ transporter	[EC:7.2.2.8]
	gene5625	copZ	K07213	copper chaperone	-
	gene0790	arsR	K03892	ArsR family transcriptional regulator, arsenate/arsenite/antimonite-responsive transcriptional repressor	-
	gene1363	arsB	K03893	arsenical pump membrane protein	-
	gene4206	arsA	K01551	arsenite/tail-anchored protein-transporting ATPase	[EC:7.3.2.7/7.3.-.-]
	gene6310	arsC	K00537	arsenate reductase (glutaredoxin)	[EC:1.20.4.1]
	gene8604	arsB	K03325	arsenite transporter	-
	gene5916	znuB	K09816	zinc transport system permease protein	-
	gene8791	znuB	K09816	zinc transport system permease protein	-
	gene5917	znuC	K09817	zinc transport system ATP-binding protein	[EC:7.2.2.20]
	gene5918	znuA	K09815	zinc transport system substrate-binding protein	-
	gene0517	moaC	K03637	cyclic pyranopterin monophosphate synthase	[EC:4.6.1.17]
	gene5137	moaC	K03637	cyclic pyranopterin monophosphate synthase [EC:4.6.1.17]	-
	gene3219	moaE	K03635	molybdopterin synthase catalytic subunit	[EC:2.8.1.12]
	gene3830	moaA	K03639	GTP 3′,8-cyclase	[EC:4.1.99.22]
	gene6814	moaA	K03639	GTP 3′,8-cyclase [EC:4.1.99.22]	-
	gene8622	moaA	K03639	GTP 3′,8-cyclase [EC:4.1.99.22]	-
	gene4835	moaD	K03636	sulfur-carrier protein	-
Carotenoid biosynthesis	gene0366	crtQ	K00514	zeta-carotene desaturase	[EC:1.3.5.6]
	gene0713	crtO	K02292	beta-carotene ketolase (CrtO type)	-
	gene1782	crtB	K02291	15-cis-phytoene synthase	[EC:2.5.1.32]
	gene2161	crtI	K10027	phytoene desaturase	[EC:1.3.99.26] [EC:1.3.99.28 ] [EC:1.3.99.29 ] [EC:1.3.99.31]
	gene4907	crtU	K09879	carotenoid phi-ring synthase/carotenoid chi-ring synthase	[EC:1.3.99.39] [EC:1.3.99.40]
	gene6428	crtP	K10210	diapolycopene oxygenase	[EC:1.14.99.44]
	gene7872	cruC	K14597	chlorobactene glucosyltransferase	-

**Table A10:** Genes involved in secretory systems predicted for *Streptomyces* sp. GD-4.

Activity Description	Gene ID	KO Name	KO ID	KO Description	Enzyme
General secretory (Sec)	gene3731	secY	K03076	preprotein translocase subunit SecY	-
	gene3781	secE	K03073	preprotein translocase subunit SecE	-
	gene5351	secA	K03070	preprotein translocase subunit SecA	[EC:7.4.2.8]
	gene6638	secG	K03075	preprotein translocase subunit SecG	-
	gene7245	secD	K03072	preprotein translocase subunit SecD	-
	gene7246	secF	K03074	preprotein translocase subunit SecF	-
	gene8418	secDF	K12257	SecD/SecF fusion protein	-
Twin-arginine translocation system	gene2382	tatA	K03116	sec-independent protein translocase protein TatA	-
	gene7067	tatA	K03116	sec-independent protein translocase protein TatA	-
	gene8221	tatA	K03116	sec-independent protein translocase protein TatA	-
	gene3274	tatB	K03117	sec-independent protein translocase protein TatB	-
	gene5174	tatD	K03424	TatD DNase family protein	[EC:3.1.21.-]
	gene7068	tatC	K03118	sec-independent protein translocase protein TatC	-

## References

[1] Hu G., Yu L., Dong Z., Lu J., Li J., Wang Y., Lai Z. (2018). Holocene Aeolian Activity in the Zoige Basin, Northeastern Tibetan Plateau, China. CATENA.

[2] Zhu Q., Chen H., Peng C., Liu J., Piao S., He J.-S., Wang S., Zhao X., Zhang J., Fang X. (2023). An Early Warning Signal for Grassland Degradation on the Qinghai-Tibetan Plateau. Nat. Commun..

[3] Xia Y., He R., Xu W., Zhang J. (2023). The Zoige Pioneer Plant *Leymus secalinus* Has Different Endophytic Bacterial Community Structures to Adapt to Environmental Conditions. PeerJ.

[4] Kang J., Zhao W., Zhao M. (2017). Remediation of Blowouts by Clonal Plants in Maqu Degraded Alpine Grasslands of Northwest China. J. Plant Res..

[5] Ghoreshizadeh S., Calvo-Peña C., Ruiz-Muñoz M., Otero-Suárez R., Coque J.J.R., Cobos R. (2024). Pseudomonas Taetrolens ULE-PH5 and *Pseudomonas* sp. ULE-PH6 Isolated from the Hop Rhizosphere Increase Phosphate Assimilation by the Plant. Plants.

[6] Myo E.M., Ge B., Ma J., Cui H., Liu B., Shi L., Jiang M., Zhang K. (2019). Indole-3-Acetic Acid Production by Streptomyces Fradiae NKZ-259 and Its Formulation to Enhance Plant Growth. BMC Microbiol..

[7] Chieb M., Gachomo E.W. (2023). The Role of Plant Growth Promoting Rhizobacteria in Plant Drought Stress Responses. BMC Plant Biol..

[8] Lu P., Hao T., Li X., Wang H., Zhai X., Tian Q., Bai W., Stevens C., Zhang W. (2021). Ambient Nitrogen Deposition Drives Plant-diversity Decline by Nitrogen Accumulation in a Closed Grassland Ecosystem. J. Appl. Ecol..

[9] Moreau D., Bardgett R.D., Finlay R.D., Jones D.L., Philippot L. (2019). A Plant Perspective on Nitrogen Cycling in the Rhizosphere. Funct. Ecol..

[10] Kane J.L., Schartiger R.G., Daniels N.K., Freedman Z.B., McDonald L.M., Skousen J.G., Morrissey E.M. (2023). Bioenergy Crop Miscanthus x Giganteus Acts as an Ecosystem Engineer to Increase Bacterial Diversity and Soil Organic Matter on Marginal Land. Soil Biol. Biochem..

[11] Zhu X., Liu D., Yin H. (2021). Roots Regulate Microbial N Processes to Achieve an Efficient NH4+ Supply in the Rhizosphere of Alpine Coniferous Forests. Biogeochemistry.

[12] Arsyadi A., Guo Y., Ebihara A., Sakagami N., Sakoda M., Tago K., Kamijo T., Ohta H., Nishizawa T. (2023). A Nitrate-Transforming Bacterial Community Dominates in the Miscanthus Rhizosphere on Nitrogen-Deficient Volcanic Deposits of Miyake-Jima. Microorganisms.

[13] Wan Y., Du Q., Wu Y., Li R., Yan X., Li N., Wang X. (2023). Rapid Dissimilatory Nitrate Reduction to Ammonium Conserves Bioavailable Nitrogen in Organic Deficient Soils. Soil Biol. Biochem..

[14] Ren H., Xu Z., Isbell F., Huang J., Han X., Wan S., Chen S., Wang R., Zeng D.-H., Jiang Y. (2017). Exacerbated Nitrogen Limitation Ends Transient Stimulation of Grassland Productivity by Increased Precipitation. Ecol. Monogr..

[15] Kraft B., Tegetmeyer H.E., Sharma R., Klotz M.G., Ferdelman T.G., Hettich R.L., Geelhoed J.S., Strous M. (2014). The Environmental Controls That Govern the End Product of Bacterial Nitrate Respiration. Science.

[16] Cameron K.C., Di H.J., Moir J.L. (2013). Nitrogen Losses from the Soil/Plant System: A Review. Ann. Appl. Biol..

[17] Kuypers M.M.M., Marchant H.K., Kartal B. (2018). The Microbial Nitrogen-Cycling Network. Nat. Rev. Microbiol..

[18] Pandey C.B. (2020). DNRA: A Short-Circuit in Biological N-Cycling to Conserve Nitrogen in Terrestrial Ecosystems. Sci. Total Environ..

[19] Hamada M.A., Soliman E.R.S. (2023). Characterization and Genomics Identification of Key Genes Involved in Denitrification-DNRA-Nitrification Pathway of Plant Growth-Promoting Rhizobacteria (Serratia Marcescens OK482790). BMC Microbiol..

[20] Li Y. (2024). Enhancing Wheat Yield Through Microbial Organic Fertilizer Substitution for Partial Chemical Fertilization: Regulation of Nitrogen Conversion and Utilization. J. Soil Sci. Plant Nutr..

[21] Moon N.J., Henk W.G. (1980). Progression of Epiphytic Microflora in Wheat and Alfalfa Silages as Observed by Scanning Electron Microscopy. Appl. Environ. Microbiol..

[22] Han C., Shi C., Liu L., Han J., Yang Q., Wang Y., Li X., Fu W., Gao H., Huang H. (2024). Majorbio Cloud 2024: Update Single-cell and Multiomics Workflows. iMeta.

[23] Wick R.R., Judd L.M., Gorrie C.L., Holt K.E. (2017). Unicycler: Resolving Bacterial Genome Assemblies from Short and Long Sequencing Reads. PLoS Comput. Biol..

[24] Besemer J., Borodovsky M. (2005). GeneMark: Web Software for Gene Finding in Prokaryotes, Eukaryotes and Viruses. Nucleic Acids Res..

[25] Kollmar M. (2019). Gene Prediction: Methods and Protocols.

[26] Van Dommelen A., Keijers V., Somers E., Vanderleyden J. (2002). Cloning and Characterisation of the Azospirillum Brasilense glnD Gene and Analysis of a glnD Mutant. Mol. Gen. Genom..

[27] Tremblay P., Hallenbeck P.C. (2009). Of Blood, Brains and Bacteria, the Amt/Rh Transporter Family: Emerging Role of Amt as a Unique Microbial Sensor. Mol. Microbiol..

[28] Forchhammer K., Selim K.A., Huergo L.F. (2022). New Views on PII Signaling: From Nitrogen Sensing to Global Metabolic Control. Trends Microbiol..

[29] Kompaniiets D., He L., Wang D., Zhou W., Yang Y., Hu Y., Liu B. (2024). Structural Basis for Transcription Activation by the Nitrate-Responsive Regulator NarL. Nucleic. Acids. Res..

[30] De Boer A.P.N., Van Der Oost J., Reijnders W.N.M., Westerhoff H.V., Stouthamer A.H., Van Spanning R.J.M. (1996). Mutational Analysis of the *Nor* Gene Cluster Which Encodes Nitric-Oxide Reductase from *Paracoccus Denitrificans*. Eur. J. Biochem..

[31] Thakur M., Anand A. (2021). Hydrogen sulfide: An Emerging Signaling Molecule Regulating Drought Stress Response in Plants. Physiol. Plant..

[32] Jaiswal S., Singh S.P., Singh S., Gupta R., Tripathi D.K., Corpas F.J., Singh V.P. (2024). Hydrogen Sulphide: A Key Player in Plant Development and Stress Resilience. Plant Cell Environ..

[33] Khan M.S.S., Islam F., Ye Y., Ashline M., Wang D., Zhao B., Fu Z.Q., Chen J. (2022). The Interplay between Hydrogen Sulfide and Phytohormone Signaling Pathways under Challenging Environments. Int. J. Mol. Sci..

[34] Wang D., Sun L., Yin Z., Cui S., Huang W., Xie Z. (2022). Insights into Genomic Evolution and the Potential Genetic Basis of *Klebsiella variicola* subsp. Variicola ZH07 Reveal Its Potential for Plant Growth Promotion and Autotoxin Degradation. Microbiol. Spectr..

[35] Liu G., Chater K.F., Chandra G., Niu G., Tan H. (2013). Molecular Regulation of Antibiotic Biosynthesis in Streptomyces. Microbiol. Mol. Biol. Rev..

[36] Mavrodi D.V., Bonsall R.F., Delaney S.M., Soule M.J., Phillips G., Thomashow L.S. (2001). Functional Analysis of Genes for Biosynthesis of Pyocyanin and Phenazine-1-Carboxamide from *Pseudomonas aeruginosa* PAO1. J. Bacteriol..

[37] Subhadra B., Surendran S., Lim B.R., Yim J.S., Kim D.H., Woo K., Kim H.-J., Oh M.H., Choi C.H. (2020). The Osmotic Stress Response Operon betIBA Is under the Functional Regulation of BetI and the Quorum-Sensing Regulator AnoR in *Acinetobacter nosocomialis*. J. Microbiol..

[38] Fourie K.R., Wilson H.L. (2020). Understanding GroEL and DnaK Stress Response Proteins as Antigens for Bacterial Diseases. Vaccines.

[39] (2016). Istamycin Aminoglycosides Profiling and Their Characterization in Streptomyces Tenjimariensis ATCC 31603 Culture Using High-performance Liquid Chromatography with Tandem Mass Spectrometry. J. Sep. Sci..

[40] Kaewkla O., Perkins M., Thamchaipenet A., Saijuntha W., Sukpanoa S., Suriyachadkun C., Chamroensaksri N., Chumroenphat T., Franco C.M.M. (2024). Description of Streptomyces Naphthomycinicus Sp. Nov., an Endophytic Actinobacterium Producing Naphthomycin A and Its Genome Insight for Discovering Bioactive Compounds. Front. Microbiol..

[41] Nofiani R., Rudiyansyah, Ardiningsih P., Rizky, Zahra S.T.A., Sukito A., Weisberg A.J., Chang J.H., Mahmud T. (2023). Genome Features and Secondary Metabolite Potential of the Marine Symbiont Streptomyces Sp. RS2. Arch. Microbiol..

[42] Iftime D., Kulik A., Härtner T., Rohrer S., Niedermeyer T.H.J., Stegmann E., Weber T., Wohlleben W. (2016). Identification and Activation of Novel Biosynthetic Gene Clusters by Genome Mining in the Kirromycin Producer *Streptomyces collinus* Tü 365. J. Ind. Microbiol. Biotechnol..

[43] Bursy J., Kuhlmann A.U., Pittelkow M., Hartmann H., Jebbar M., Pierik A.J., Bremer E. (2008). Synthesis and Uptake of the Compatible Solutes Ectoine and 5-Hydroxyectoine by *Streptomyces coelicolor* A3(2) in Response to Salt and Heat Stresses. Appl. Environ. Microbiol..

[44] Seyedsayamdost M.R., Traxler M.F., Zheng S.-L., Kolter R., Clardy J. (2011). Structure and Biosynthesis of Amychelin, an Unusual Mixed-Ligand Siderophore from *Amycolatopsis* sp. AA4. J. Am. Chem. Soc..

[45] Bhattacharyya P.N., Jha D.K. (2012). Plant Growth-Promoting Rhizobacteria (PGPR): Emergence in Agriculture. World J. Microbiol. Biotechnol..

[46] De Andrade L.A., Santos C.H.B., Frezarin E.T., Sales L.R., Rigobelo E.C. (2023). Plant Growth-Promoting Rhizobacteria for Sustainable Agricultural Production. Microorganisms.

[47] Gupta R., Khan F., Alqahtani F.M., Hashem M., Ahmad F. (2024). Plant Growth–Promoting Rhizobacteria (PGPR) Assisted Bioremediation of Heavy Metal Toxicity. Appl. Biochem. Biotechnol..

[48] Narsing Rao M.P., Lohmaneeratana K., Bunyoo C., Thamchaipenet A. (2022). Actinobacteria–Plant Interactions in Alleviating Abiotic Stress. Plants.

[49] Zhu X., Meng L., Jiang C., Nie W., Cao Y., Lian B. (2024). The Mechanism of a Multifunctional Strain of *Streptomyces* sp. on the Growth of *Pinus massoniana* Seedlings. J. Soil Sci. Plant Nutr..

[50] Youseif S.H., El-Megeed F.H.A., Salous M.S., Mohamed A.H. (2023). *Streptomyces* Biostimulants: An Effective Sustainable Approach to Reduce Inorganic N Input and Maintain High Yield of Wheat Crop in Different Soil Types. J. Appl. Microbiol..

[51] Qin S., Feng W.-W., Wang T.-T., Ding P., Xing K., Jiang J.-H. (2017). Plant Growth-Promoting Effect and Genomic Analysis of the Beneficial Endophyte *Streptomyces* sp. KLBMP 5084 Isolated from Halophyte Limonium Sinense. Plant Soil.

[52] Boukelloul I., Aouar L., Cherb N., Carvalho M.F., Oliveira R.S., Akkal S., Nieto G., Zellagui A., Necib Y. (2024). Actinobacteria Isolated from Soils of Arid Saharan Regions Display Simultaneous Antifungal and Plant Growth Promoting Activities. Curr. Microbiol..

[53] Zhu H., Hu L., Hu H., Zhou F., Wu L., Wang S., Rozhkova T., Li C. (2023). Identification of a Novel *Streptomyces* Sp. Strain HU2014 Showing Growth Promotion and Biocontrol Effect Against *Rhizoctonia* spp. in Wheat. Plant Dis..

[54] Nonthakaew N., Panbangred W., Songnuan W., Intra B. (2022). Plant Growth-Promoting Properties of *Streptomyces* spp. Isolates and Their Impact on Mung Bean Plantlets’ Rhizosphere Microbiome. Front. Microbiol..

[55] Stephens B.B., Loar S.N., Alexandre G. (2006). Role of CheB and CheR in the Complex Chemotactic and Aerotactic Pathway of *Azospirillum brasilense*. J. Bacteriol..

[56] Kanungpean D., Kakuda T., Takai S. (2011). Participation of CheR and CheB in the Chemosensory Response of Campylobacter Jejuni. Microbiology.

[57] García-Fontana C., Reyes-Darias J.A., Muñoz-Martínez F., Alfonso C., Morel B., Ramos J.L., Krell T. (2013). High Specificity in CheR Methyltransferase Function. J. Biol. Chem..

[58] Augimeri R.V., Varley A.J., Strap J.L. (2015). Establishing a Role for Bacterial Cellulose in Environmental Interactions: Lessons Learned from Diverse Biofilm-Producing Proteobacteria. Front. Microbiol..

[59] Zhang X., Wu J., Kong Z. (2024). Cellular Basis of Legume–Rhizobium Symbiosis. Plant Commun..

[60] Zhang Y., Ku Y.-S., Cheung T.-Y., Cheng S.-S., Xin D., Gombeau K., Cai Y., Lam H.-M., Chan T.-F. (2024). Challenges to Rhizobial Adaptability in a Changing Climate: Genetic Engineering Solutions for Stress Tolerance. Microbiol. Res..

[61] Frungillo L. (2022). Getting to the Root of Nodulation: How Legumes and Rhizobia Use Nitrate Uptake to Control Symbiosis. Plant Cell.

[62] Koch H. (2024). The Microbial-Driven Nitrogen Cycle and Its Relevance for Plant Nutrition. J. Exp. Bot..

[63] Putz M., Schleusner P., Rütting T., Hallin S. (2018). Relative Abundance of Denitrifying and DNRA Bacteria and Their Activity Determine Nitrogen Retention or Loss in Agricultural Soil. Soil Biol. Biochem..

[64] Wei Z. (2022). Biochar Amendment Alters the Partitioning of Nitrate Reduction by Significantly Enhancing DNRA in a Paddy Field. Biochar.

[65] Friedl J., De Rosa D., Rowlings D.W., Grace P.R., Müller C., Scheer C. (2018). Dissimilatory Nitrate Reduction to Ammonium (DNRA), Not Denitrification Dominates Nitrate Reduction in Subtropical Pasture Soils upon Rewetting. Soil Biol. Biochem..

[66] Huang X., Luoluo, Xie D., Li Z. (2023). Dissimilatory Nitrate Reduction to Ammonium in Four *Pseudomonas* spp. under Aerobic Conditions. Heliyon.

[67] Reay M.K., Marsden K.A., Powell S., Rivera L.M., Chadwick D.R., Jones D.L., Evershed R.P. (2023). The Soil Microbial Community and Plant Biomass Differentially Contribute to the Retention and Recycling of Urinary-N in Grasslands. Soil Biol. Biochem..

[68] Makino K., Shinagawa H., Amemura M., Kawamoto T., Yamada M., Nakata A. (1989). Signal Transduction in the Phosphate Regulon of *Escherichia coli* Involves Phosphotransfer between PhoR and PhoB Proteins. J. Mol. Biol..

[69] Eichhorn E., Van Der Ploeg J.R., Leisinger T. (2000). Deletion Analysis of the *Escherichia coli* Taurine and Alkanesulfonate Transport Systems. J. Bacteriol..

[70] Tang J., Li Y., Zhang L., Mu J., Jiang Y., Fu H., Zhang Y., Cui H., Yu X., Ye Z. (2023). Biosynthetic Pathways and Functions of Indole-3-Acetic Acid in Microorganisms. Microorganisms.

[71] Salwan R., Sharma V., Sharma A., Singh A. (2020). Molecular Imprints of Plant Beneficial *Streptomyces* sp. AC30 and AC40 Reveal Differential Capabilities and Strategies to Counter Environmental Stresses. Microbiol. Res..

[72] Etesami H., Glick B.R. (2024). Bacterial Indole-3-Acetic Acid: A Key Regulator for Plant Growth, Plant-Microbe Interactions, and Agricultural Adaptive Resilience. Microbiol. Res..

[73] Cen X., Li H., Zhang Y., Huang L., Luo Y. (2024). Isolation and Plant Growth Promotion Effect of Endophytic Siderophore-Producing Bacteria: A Study on Halophyte Sesuvium Portulacastrum. Plants.

[74] Timofeeva A.M., Galyamova M.R., Sedykh S.E. (2023). Plant Growth-Promoting Soil Bacteria: Nitrogen Fixation, Phosphate Solubilization, Siderophore Production, and Other Biological Activities. Plants.

[75] Sun Y. (2022). Screening of Siderophore-Producing Bacteria and Their Effects on Promoting the Growth of Plants. Curr. Microbiol..

[76] Codd R., Richardson-Sanchez T., Telfer T.J., Gotsbacher M.P. (2018). Advances in the Chemical Biology of Desferrioxamine B. ACS Chem. Biol..

[77] Wang W., Qiu Z., Tan H., Cao L. (2014). Siderophore Production by Actinobacteria. Biometals.

[78] Cruz-Morales P., Ramos-Aboites H.E., Licona-Cassani C., Selem-Mójica N., Mejía-Ponce P.M., Souza-Saldívar V., Barona-Gómez F. (2017). Actinobacteria Phylogenomics, Selective Isolation from an Iron Oligotrophic Environment and Siderophore Functional Characterization, Unveil New Desferrioxamine Traits. FEMS Microbiol. Ecol..

[79] Araujo R., Dunlap C., Barnett S., Franco C.M.M. (2019). Decoding Wheat Endosphere–Rhizosphere Microbiomes in Rhizoctonia Solani–Infested Soils Challenged by Streptomyces Biocontrol Agents. Front. Plant Sci..

[80] Zhang Y., Zhang T., Xue Z., Liu Y., Li Y., Li Y., Chen Q. (2021). Streptomyces Application Triggers Reassembly and Optimization of the Rhizosphere Microbiome of Cucumber. Diversity.

[81] Hu D., Li S., Li Y., Peng J., Wei X., Ma J., Zhang C., Jia N., Wang E., Wang Z. (2020). *Streptomyces* sp. Strain TOR3209: A Rhizosphere Bacterium Promoting Growth of Tomato by Affecting the Rhizosphere Microbial Community. Sci. Rep..

[82] Richter A.A., Mais C.-N., Czech L., Geyer K., Hoeppner A., Smits S.H.J., Erb T.J., Bange G., Bremer E. (2019). Biosynthesis of the Stress-Protectant and Chemical Chaperon Ectoine: Biochemistry of the Transaminase EctB. Front. Microbiol..

[83] Bagnoli F., Rappuoli R. (2017). Protein and Sugar Export and Assembly in Gram-Positive Bacteria.

[84] Tseng T.-T., Tyler B.M., Setubal J.C. (2009). Protein Secretion Systems in Bacterial-Host Associations, and Their Description in the Gene Ontology. BMC Microbiol..

[85] Wei M., Jiao M., Nie X., Wang C., Yu X., Liu Y., Wei X. (2024). Genomic and Metabolomic Profiling Reveal *Streptomyces rochei* S32 Contributes to Plant Growth by Nitrogen Fixation and Production of Bioactive Substances. Plant Soil.

[86] Wang Q., Zhao J., Liu Z., Ding S., Huang Z., Chen J. (2024). Genomic Insights and Synthetic Biology Applications of Marine Actinomycete Streptomyces Griseoincarnatus HNS054. Int. J. Mol. Sci..

[87] Fatima A., Abbas M., Nawaz S., Rehman Y., Rehman S.U., Sajid I. (2024). Whole Genome Sequencing (WGS) and Genome Mining of *Streptomyces* sp. AFD10 for Antibiotics and Bioactive Secondary Metabolites Biosynthetic Gene Clusters (BGCs). Gene Rep..

[88] Dávila Costa J.S., Hoskisson P.A., Paterlini P., Romero C.M., Alvarez A. (2020). Whole Genome Sequence of the Multi-Resistant Plant Growth-Promoting Bacteria *Streptomyces* sp. Z38 with Potential Application in Agroindustry and Bio-Nanotechnology. Genomics.

[89] Xu G., Wu W., Zhu L., Liang Y., Liang M., Tan S., Chen H., Huang X., He C., Lu Y. (2024). Whole Genome Sequencing and Biocontrol Potential of Streptomyces Luteireticuli ASG80 Against Phytophthora Diseases. Microorganisms.

[90] Vaz Jauri P., Beracochea M., Fernández B., Battistoni F. (2019). Whole-Genome Sequencing of *Streptomyces* sp. Strain UYFA156, a Cultivar-Specific Plant Growth-Promoting Endophyte of *Festuca Arundinacea*. Microbiol. Resour. Announc..

[91] Gallegos-Lopez S., Mejia-Ponce P.M., Gonzalez-Salazar L.A., Rodriguez-Orduña L., Souza-Saldivar V., Licona-Cassani C. (2020). Draft Genome Sequence of *Streptomyces* sp. Strain C8S0, Isolated from a Highly Oligotrophic Sediment. Microbiol. Resour. Announc..

[92] Jiang L., Zeng Z., Wang Z., Tang M., Jiang S., Ma Q., Wang Z., Peng D., Li S., Pu H. (2024). Genomic Investigation of a Rhizosphere Isolate, *Streptomyces* sp. JL1001, Associated with Polygonatum Cyrtonema Hua. Curr. Microbiol..

[93] Duangupama T., Pittayakhajonwut P., Intaraudom C., Suriyachadkun C., Tadtong S., Kuncharoen N., He Y.-W., Tanasupawat S., Thawai C. (2024). Description of Streptomyces *Siderophoricus* sp. nov., a Promising Nocardamine-Producing Species Isolated from the Rhizosphere Soil of Mangifera Indica. J. Antibiot..

[94] Li L.-F., Wu Q.-X., Wu H., Li Y., Peng Q., Han R.-H., Zhang D.-H., Yu W.-D., Xu R., Wang J. (2023). Complete Genome Sequence of Streptomyces Sp. HP-A2021, a Promising Bacterium for Natural Product Discovery. Biochem. Genet..

